# Macrophage DCLK1 promotes obesity-induced cardiomyopathy via activating RIP2/TAK1 signaling pathway

**DOI:** 10.1038/s41419-023-05960-4

**Published:** 2023-07-13

**Authors:** Bin Yang, Yunjie Zhao, Wu Luo, Weiwei Zhu, Leiming Jin, Minxiu Wang, Lin Ye, Yi Wang, Guang Liang

**Affiliations:** 1 Department of Pharmacy and Institute of Inflammation, Zhejiang Provincial People’s Hospital, Affiliated People’s Hospital, Hangzhou Medical College, Hangzhou, Zhejiang 310014 China; 2 Chemical Biology Research Center, School of Pharmaceutical Sciences, Wenzhou Medical University, Wenzhou, Zhejiang 325035 China; 3 Medical Research Center, the First Affiliated Hospital, Wenzhou Medical University, Wenzhou, Zhejiang 325035 China

**Keywords:** Molecular biology, Immunology

## Abstract

Obesity increases the risk for cardiovascular diseases and induces cardiomyopathy. Chronic inflammation plays a significant role in obesity-induced cardiomyopathy and may provide new therapeutic targets for this disease. Doublecortin-like kinase 1 (DCLK1) is an important target for cancer therapy and the role of DCLK1 in obesity and cardiovascular diseases is unclear. Herein, we showed that DCLK1 was overexpressed in the cardiac tissue of obese mice and investigated the role of DCLK1 in obesity-induced cardiomyopathy. We generated DCLK1-deleted mice and showed that macrophage-specific DCLK1 knockout, rather than cardiomyocyte-specific DCLK1 knockout, prevented high-fat diet (HFD)-induced heart dysfunction, cardiac hypertrophy, and fibrosis. RNA sequencing analysis showed that DCLK1 deficiency exerted cardioprotective effects by suppressing RIP2/TAK1 activation and inflammatory responses in macrophages. Upon HFD/palmitate (PA) challenge, macrophage DCLK1 mediates RIP2/TAK1 phosphorylation and subsequent inflammatory cytokine release, which further promotes hypertrophy in cardiomyocytes and fibrogenesis in fibroblasts. Finally, a pharmacological inhibitor of DCLK1 significantly protects hearts in HFD-fed mice. Our study demonstrates a novel role and a pro-inflammatory mechanism of macrophage DCLK1 in obesity-induced cardiomyopathy and identifies DCLK1 as a new therapeutic target for the treatment of this disease.

**Figure:**
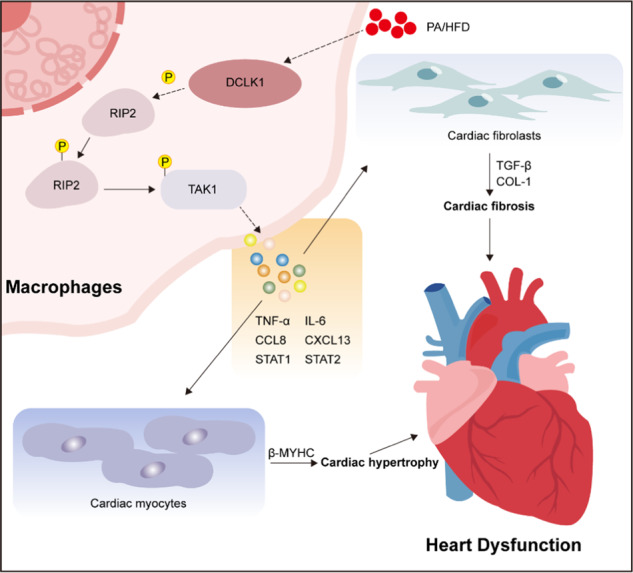
Upon HFD/PA challenge, DCLK1 induces RIP2/TAK1-mediated inflammatory response in macrophages, which subsequently promotes cardiac hypertrophy and fibrosis. Macrophage-specific DCLK1 deletion or pharmacological inhibition of DCLK1 protects hearts in HFD-fed mice.

Upon HFD/PA challenge, DCLK1 induces RIP2/TAK1-mediated inflammatory response in macrophages, which subsequently promotes cardiac hypertrophy and fibrosis. Macrophage-specific DCLK1 deletion or pharmacological inhibition of DCLK1 protects hearts in HFD-fed mice.

## Introduction

Obesity, described as a body mass index ≥30 kg/m^2^, is related to an elevated risk of multiple major reasons for preventing death, such as cardiovascular disorders, metabolic syndrome, type 2 diabetes, and some forms of cancer [1–4]. Obesity generates cardiac remodeling and impairs heart functions, resulting in a changed hemodynamic load, myocardial fibrosis, and reduced ventricular contraction ability, which ultimately leads to heart failure [5–8]. A multitude of cellular and molecular mechanisms, including adipose tissue dysfunction, systemic inflammation, metabolic disturbances, oxidative stress, autophagy/mitophagy defects, myocardial fibrosis, and endothelial impairment, are implicated in the causality of obesity-induced cardiomyopathy, according to recent research [9]. Chronic inflammation performs a major part in the pathogenesis of cardiomyopathy associated with obesity [10]. Macrophages are essential constituents of innate immunity and regulate myocardial inflammation and remodeling in a variety of ways [11]. By producing pro-inflammatory mediators, such as TNF-α, IL-1β, and IL-6, macrophages induce pathological cardiac hypertrophy and disrupt the systolic and diastolic functioning of the heart [12–14]. Monocytes generated from bone marrow infiltrate the heart following damage to enhance the pathophysiology and progression of obesity-induced cardiac harm [15]. Novel treatment techniques are required to consistently avoid the onset of heart disorders associated with obesity.

Doublecortin-like kinase 1 (DCLK1) is a transmembrane microtubule-related protein kinase originally observed in the nervous system where it regulates microtubule polymerization and promotes neuronal migration [16]. Later studies showed that DCLK1 is a novel specific surface marker of tumor stem cells and it shows an increase in several human malignant tumors. DCLK1 serves a major regulatory involvement in cancer aggressiveness [17, 18]. Houchen et al. reported that pharmacological inhibition of DCLK1 exerts anticancer effect on colorectal and pancreatic cancer cells [19]. Thus, DCLK1 is a crucial therapeutic target for treating cancer. Although there is a plethora of information on neuro-regenerative and cancer-promoting impacts of DCLK1, few research has been conducted on the involvement of DCLK1 in the onset of non-malignant illnesses. Studies suggest that DCLK1 mediates the response to inflammatory factors, enhances inflammation-induced epithelial repair, and reduces chronic colitis [20]. Reports also suggest that DCLK1 may contribute significantly to the development of hepatitis C virus-induced chronic liver disease [21]. However, the role of DCLK1 in obesity and cardiovascular diseases has not yet been studied.

Our group has been engaged in finding new inflammatory mechanism and targets in diabetic and obese cardiomyopathy for many years [22–24]. The background check on DCLK1 inspired us to examine DCLK1 expression change in the heart tissues of high-fat diet (HFD)-fed mice, which were originally used in other projects of our group. Interestingly, we found that DCLK1 is overexpressed in the cardiac tissue of obese mice. Then, we investigated the role of DCLK1 in obesity-induced cardiomyopathy. The present findings showed that DCLK1 in macrophages, rather than DCLK1 in cardiomyocytes, mediates chronic inflammation leading to subsequent damage to the cardiac tissue of obese mice. Pharmacological inhibition or macrophage-specific knockout of DCLK1 had a protective effect on the cardiac tissue of HFD-fed mice. Our research demonstrates a novel role of DCLK1 and identifies DCLK1 as a new treatment target for obesity-induced cardiomyopathy.

## Materials and methods

### Reagents and cell culture

We isolated the neonatal rat cardiomyocytes (NRCMs) and neonatal rat cardiac fibroblasts (NRCFs) using a previously published protocol [25]. Briefly, neonatal rats were sacrificed. Heart tissues were removed, washed in PBS, and digested using a mixture of pancreatin and collagenase. NRCFs were removed by differential adherence culture and cultured in DMEM medium containing 4.5 g/L glucose and 10% fetal bovine serum (FBS). Myocardium cells were also cultured in the same media with the addition of 5-BrdU. After 48 hours of culture, NRCMs exhibited regular pulsation and were used for experiments.

Primary mouse peritoneal macrophages (MPMs) were prepared from C57BL/6 wildtype mice. Mice were administered 1 ml of 4% thioglycolate in PBS intraperitoneally. Three days later, peritoneal cells were collected and incubated in DMEM/F12 supplemented with 10% FBS for 4 hours. Cells were then incubated at 37 °C for 6 h and washed with PBS to remove the non-adherent cells. The remaining adherent cells were used as the peritoneal macrophages described in the experiments. A list of the reagents used in this experiment is shown in Supplemental Table S1.

### Animal experiments

Wildtype C57BL/6, B6/JGpt-Dclk1em1Cflox/Gpt, B6/JGpt-Lyz2em1Cin(iCre)/Gpt, and B6/JGpt-H11em1Cin (Myh6-iCre)/Gpt were obtained from GemPharmatech Co. Ltd (Nanjing, China) (Supplemental Table S1). Using the Dclk1^flox^ mice and respective Cre mice, macrophage-specific DCLK1 knockout mice and cardiomyocyte-specific DCLK1 knockout mice were generated with technical expertize from GemPharmatech Co. Ltd (Nanjing, China). The genotyping data of macrophage-specific DCLK1 knockout mice and cardiomyocyte-specific DCLK1 knockout mice using genomic DNA isolated from tail clip samples were shown in the Supplementary Fig. S1. All of the mice were kept at the Wenzhou Medical University Animal Center, which is a pathogen-free facility. They were kept in cages with a temperature of 23 ± 2 °C., with a light/dark cycle, and access to free food and drink at all times. All caring and trial methods were accepted by the Animal Policy and Welfare Committee at Wenzhou Medical University (No. wydw2018-0224), and all mouse models adhered to the rules established by the National Institutes of Health (NIH) (Guide for the care and use of laboratory animals). Animal studies are reported in compliance with the ARRIVE guidelines (The ARRIVE guidelines 2.0).

1. HFD-induced obesity mice given DCLK1 inhibitor DCLK1-IN-1 (5 and 10 mg/kg). For therapeutic suppression, wildtype C57BL/6 mice were randomly separated into two weight-matched groups: (i) a low-fat diet (LFD group, *n* = 6) or (ii) a high-fat diet group (HFD, *n* = 18) for 12 weeks. Six control mice were provided with a regular rodent diet. The standard control meal (having 10 kcal% fat, 20 kcal% protein, and 70 kcal% carbs) while HFD (having 60 kcal% fat, 20 kcal% protein, and 20 kcal% carbs) were bought from Medicience Diets Co. LTD, Yangzhou, China. Following 12 weeks, the HFD group mice were randomly separated into three groups. (i) HFD-induced obese mice (HFD group, *n* = 6), given vehicle for DCLK1-IN, (ii) HFD-induced obesity mice given DCLK1-IN 5 mg/kg (DCLK1-IN-5 + HFD group, *n* = 6), (iii) HFD-induced obesity mice given DCLK1-IN 10 mg/kg (DCLK1-IN-10 + HFD group, *n* = 6). HFD mice were given DCLK1-IN orally once every two days from the 13th week to the 20th week. Mice in control and HFD groups were orally given the vehicle on a similar frequency. All mice were euthanized by anesthesia at the completion of the 20th week, the end body weight was determined and the hearts and blood samples were recovered.
2. HFD-induced obesity mice with macrophage-specific DCLK1 knockout: We crossed DCLK1 flox mice with Lyz-Cre to knockout DCLK1 in myeloid cell lineage, such as monocytes, mature macrophages, and granulocytes. Four groups were examined: DCLK1^f/f^ -LFD, DCLK1^lyz-cre^ -LFD, DCLK1^f/f^ -HFD, and DCLK1^lyz-cre^ -HFD (*n* = 6 per group).
3. HFD-induced obesity mice with cardiomyocyte-specific DCLK1 knockout: The cardiomyocyte-specific DCLK1 knockout mice (DCLK1^Myh6-cre^) were developed utilizing the Cre-loxP approach. Mice floxed for DCLK1 (DCLK1^f/f^) were crossed with mice carrying Cre-transgene under the promoter of the Myh6 gene (Myh6-Cre) which resulted in the development of DCLK1^Myh6-cre^ mice. Four groups were examined: DCLK1^f/f^ -LFD, DCLK1^Myh6-cre^ -LFD, DCLK1^f/f^ -HFD, and DCLK1^Myh6-cre^ -HFD (*n* = 6 per group).

In all investigations, the body weights of mice were measured weekly. At the completion of the experiment, mice were sacrificed. We collected left ventricular tissue at 5 mm thick from the apex of the heart. The collection of blood samples, and the weighing and processing of cardiac tissues were conducted. Serum levels of serum creatine kinase MB (CK-MB) and ANP were utilized to evaluate the cardiac function of trial groups. Employing commercially available tests, total serum triglyceride, cholesterol, LDL-C, and HDL-C were determined. Slices of the heart’s tissues were fixed in formalin and embedded in paraffin. Tissues were also embedded in an Optimal cutting temperature (OCT) solution to prepare frozen slices. Other downstream gene and protein analyses were conducted on tissue samples that had been snap-frozen.

### Cell transfections

To express wildtype DCLK1 or RIP2 in MPMs, transfection with DCLK1 or RIP2 cDNA plasmid Flag-DCLK1 or Flag-RIP2 was performed using polyetherimide. Briefly, the transfection complex was prepared with 3:1 polyetherimide (μg) and plasmid (μg) in a 100 μL Opti MEM medium. MPMs were incubated with a transfection complex for 6 h, and then the media was changed with a new growth media.

### Assessment of heart function

For echocardiography, the mice were anesthetized with 3% isoflurane and placed on a plate without mechanical ventilation. The heart rate of mice was maintained between 480 and 520 BPM. An echocardiography examination was performed in the multi-mode small animal ultrasound imaging system (Vevo 3100; FUJIFILM Visual Sonics, Canada). The ejection fraction (EF%) was calculated by subtracting the end-diastolic volume of the left ventricle (LVEDV) from the end-systolic volume of the left ventricle (LVESV) and plugging those values into the formula ((LVEDV–LVESV)/LVEDV × 100). To determine the amount of fractional shortening (FS), a formula (FS% = [(LVIDd–LVIDs)/LVIDd] × 100 was utilized.

### Reverse transcription and real-time quantitative PCR

To recover RNA from heart tissues ranging in weight from 80–100 mg, homogenization in TRIZOL (manufactured by Invitrogen and located in Carlsbad, California, USA) was performed following the protocols provided by the supplier. We used a PrimeScriptTM RT reagent Kit with gDNA Eraser and SYBR premix Ex Taq II to conduct a reverse transcription as well as quantitative PCR (TAKARA). For the quantitative PCR analysis, a Bio-Rad CFX96 real-time system (Bio-Rad, USA) was utilized. Data were normalized to the amount of *Actb*. The primers were acquired through Invitrogen (Shanghai, China). Supplementary Table S2 lists the sequences of the primers. The 2−ΔΔCt method was utilized to determine the relative frequency of the target genes.

### Western blot and co-immunoprecipitation

Homogenates derived from cell or tissue lysates were subjected to 10% Sodium dodecyl sulfate-polyacrylamide gel electrophoresis before being electro-transferred to polyvinylidene fluoride (PVDF) membranes. Membranes were blocked for 1.5 h in a solution that consisted of Tris-buffered saline with 0.05% Tween20 and 5% non-fat milk. Following this step, PVDF membranes were treated with the proper primary antibodies. Then, the membrane was washed with Tris-Buffered saline and tween20 (TBST) and the horseradish peroxidase (HRP) conjugated secondary antibody was incubated at room temperature for one hour. Finally, the signal in the membrane activated by the chemiluminescence (ECL) reagent was detected using an ECL system. Image J analysis program (version 1.38e) was utilized to carry out densitometric quantification, and the results were then normalized to each group’s matching controls.

For co-immunoprecipitation studies, a small amount of lysate was removed as input. The remaining samples were incubated with the target antibody at 4 °C overnight. Subsequently, magnetic beads were added to the protein lysate and incubated at 4 °C for 2 hours. The supernatant was discarded after centrifugation. The magnetic bead mixture was washed with PBS and buffer containing SDS was added. The samples were used for subsequent western blot experiments.

### Immunohistochemical determination

The samples were fixed and dehydrated, in a series of graded alcohol, cleared with xylene, and embedded with paraffin. For immunohistological procedures, paraffin slices were dewaxed, rehydrated in a series of ethanol concentrations, exposed to antigen retrieval in 0.01 mol/L citrate buffer (pH 6.0) for 3 min, and incubated for 30 min at room temperature in 3% H_2_O_2_ in methanol. Following blocking with 5% bovine serum albumin (BSA), these slices were incubated for one night at 4 °C with primary antibodies (1:200) against F4/80, then incubated with the matching secondary antibodies (1:200, Cell Signaling Technology). Diaminobenzidine solution was utilized to visualize the reaction. Following hematoxylin counterstaining, slices were dried and examined under a light microscope (×200 magnification; Nikon, Japan).

### Histopathology

Preserved cardiac tissue was embedded in paraffin and spliced at 5 µm. Following dryness, slices were stained with H&E (hematoxylin and eosin) kit according to the supplier’s guidelines. Employing a light microscope (×200 magnification; Nikon, Tokyo, Japan), the histopathological lesions were assessed and documented.

### Picro-Sirius red and Masson staining for collagen

The preserved heart tissues were embedded in paraffin and dissected at a thickness of 5 µm. Picro-Sirius red, a selective stain for collagen, and Masson’s trichrome were utilized to assess collagen deposition in kidney tissue and heart tissue. Employing a light microscope (×200 magnification; Nikon, Japan), the staining slices were photographed on film. Image J (NIH) was employed to quantify collagen deposition utilizing a single-blind approach.

### WGA-FITC staining

The preserved tissues were embedded in paraffin and dissected at a thickness of 5 µm. The dewaxed and rehydrated slices were incubated for 30 min at 37 °C with the wheat germ agglutinin (WGA)-FITC. Following PBS wash (pH7.4), DAPI was applied to counterstain the nucleus. A fluorescent microscope (×200 magnification; Nikon, Japan) was employed to capture images.

### Rhodamine phalloidin staining

NRCMs were preserved with 4% paraformaldehyde, after that, permeabilized with 0.1% Triton X-100, and stained with rhodamine-conjugated phalloidin (Phalloidin-Rho) (50 ng/ml) for 30 min at room temperature to evaluate hypertrophic alterations. DAPI was applied to stain nuclei at room temperature. A Nikon epifluorescence microscope (400×; Nikon, Tokyo, Japan) was employed to observe and record immunofluorescence.

### Double immunofluorescence staining

Mice hearts that had been recovered were immersed in the OCT mixture and dissected to a thickness of 5 μm. Up overnight, slices were incubated with primary mouse antibodies against DCLK1, vimentin, α-actin, and CD68 in a humid environment at 4 °C. Thereafter, the slices were treated for 1 h in the darkness with Alexa-488 (anti-mouse) and Alexa-594 (anti-rabbit) conjugated secondary antibodies. The slices were washed with PBS three times and DAPI was applied to stain nuclei at room temperature.

### Intercellular crosstalk experiment

MPMs from DCLK1f/f and DCLK1lyz-cre mice were exposed to 200 μM PA or vehicle for 24 h. The conditioned media (CM) of above MPMs were collected. The CM from four MPM groups was supplied in a 1:1 ratio with fresh media and then incubated to NRCMs or neonatal rat cardiac fibroblasts (NRCFs). The impact of MPM CM on NRCMs was evaluated by labeling cells for cardiac hypertrophy. qPCR for activation signatures was utilized to assess NRCFs for cardiac fibrosis.

### Cytokine measurements

The levels of TNF-α and IL-6 in cell culture supernatants were measured utilizing ELISA kits following the supplier’s recommendations.

### Transcriptome sequencing

RNA was recovered and refined utilizing TRIzol (Invitrogen, CA, USA) based on the supplier’s directions. Utilizing NanoDrop ND-1000, the RNA concentration and purity of each cardiac sample were measured (NanoDrop, DE, USA). The RNA integrity was assessed using a Bioanalyzer 2100 (Agilent, CA, USA) and validated using an agarose gel electrophoresis. 1 μg of total RNA is applied to purify Poly (A) RNA utilizing Dynabeads Oligo (dT)25-61005 (Thermo Fisher, CA, USA). Thereafter, the poly(A) RNA was split into smaller fragments utilizing Magnesium RNA Fragmentation Module (NEB, cat. e6150, MA, USA). In addition, SuperScriptTM II reverse transcriptase was used to transcribe the cleaved RNA fragments into cDNA (Invitrogen, cat. 1896649, CA, USA). As per the supplier’s guidelines, the sequencing analysis was completed utilizing an Illumina NovaseqTM 6000. Following the development of the complete transcriptome, the expression patterns of all transcripts were examined. mRNAs with differential expression were chosen based on the next criteria: *P* < 0.05 and fold change > 2 or fold change < 0.5.

### Statistical analysis

All trials are conducted in blind and randomized settings. All information is reported as Mean ± SEM. The statistical analyses were conducted employing GraphPad Prism 8.0 (San Diego, CA, USA). An unpaired nonparametric 2-tailed Student’s *t* test was applied for comparing two groups, and one-way ANOVA followed by a nonparametric Tukey post hoc test was applied for comparing more than two groups. *P* < 0.05 were regarded as statistically significant. Only if F attained *P* < 0.05 and there was no significant variance inhomogeneity were post-tests conducted.

## Results

### DCLK1 is highly expressed in the heart of obese mice and is localized to cardiomyocytes and macrophages

**Fig. 1:**
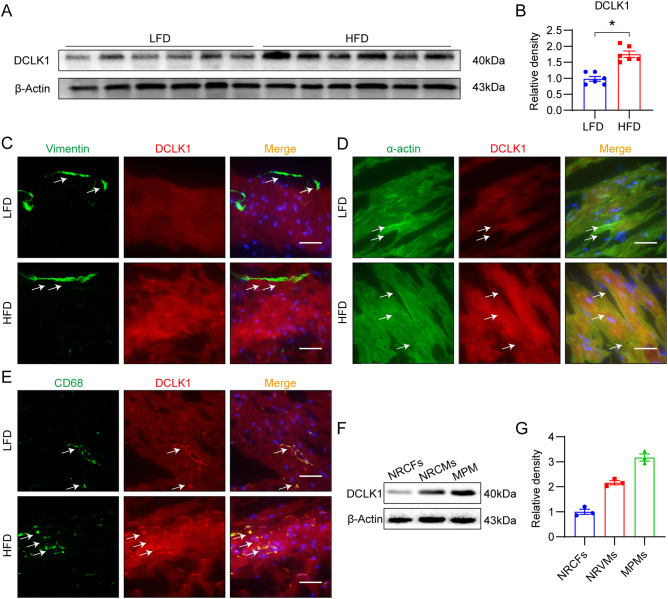
DCLK1 is upregulated in the hearts of obese mice, and is localized to cardiomyocytes and macrophages rather than fibroblasts. **A**, **B** DCLK1 protein levels in heart tissues of DCLK1^f/f^ mice fed an LFD or an HFD for 20 weeks. β-actin was used as the loading control. Densitometric quantification is shown in **B** [*n* = 6; Student’s *t* test; mean ± SEM; *p < 0.05]. **C** Representative immunofluorescence staining of heart tissues of mice. Figure showing fibroblast marker vimentin (green) and DCLK1 (red) in heart samples from mice fed LFD and HFD. Tissues were counterstained with DAPI (blue) [scale bar = 50 μm; *n* = 6]. **D** Representative immunofluorescence staining of heart tissues of mice. Figure showing cardiomyocyte marker α-actin (green) and DCLK1 (red) in heart samples from mice fed LFD and HFD. Tissues were counterstained with DAPI (blue) [scale bar = 50 μm; *n* = 6]. **E** Representative immunofluorescence staining of heart tissues of mice. Figure showing macrophage marker CD68 (green) and DCLK1 (red) in heart samples from mice fed LFD and HFD. Tissues were counterstained with DAPI (blue) [scale bar = 50 μm; *n* = 6]. **F**, **G** DCLK1 protein levels in NRCFs, NRCMs, and MPMs. Densitometric quantification is shown in **G** [*n* = 3].

To determine whether DCLK1 is involved in HFD-induced cardiac dysfunction, we analyzed its expression in the heart tissue of fat mice. Twenty weeks were spent feeding mice an HFD or a typical diet (LFD). Immunoblotting findings revealed that DCLK1 expression is elevated in the heart tissue of mice fed on HFD (Fig. 1A, B). Cardiomyocytes and fibroblasts are the two most abundant cell kinds in the heart [26]. Additionally, resident macrophages are found in the normal heart, wherein they gain the M2 phenotype; however, macrophages can adopt the inflammatory phenotype in obese conditions [12]. We performed immunostaining of the mouse heart tissue to examine the distribution of DCLK1. Results showed that DCLK1 is co-localized with the cardiomyocyte marker α-actin and macrophage marker CD68, but not with the cardiac fibroblast marker vimentin (Fig. 1C–E). Increased DCLK1 expression in the heart tissue was coupled with a rise in macrophage infiltration of the heart in HFD-fed mice. Consistently, western blot analysis using isolated primary cells showed that DCLK1 is mainly expressed in mouse primary macrophages (MPMs) and NRCMs under the basal condition (Fig. 1F, G). Collectively, these findings revealed that DCLK1 showed its expression in cardiomyocytes as well as macrophages, and might be associated with the pathogenesis of obesity-induced cardiomyopathy.

### Macrophage-specific DCLK1 knockout prevents HFD-induced cardiac hypertrophy and fibrosis

**Fig. 2:**
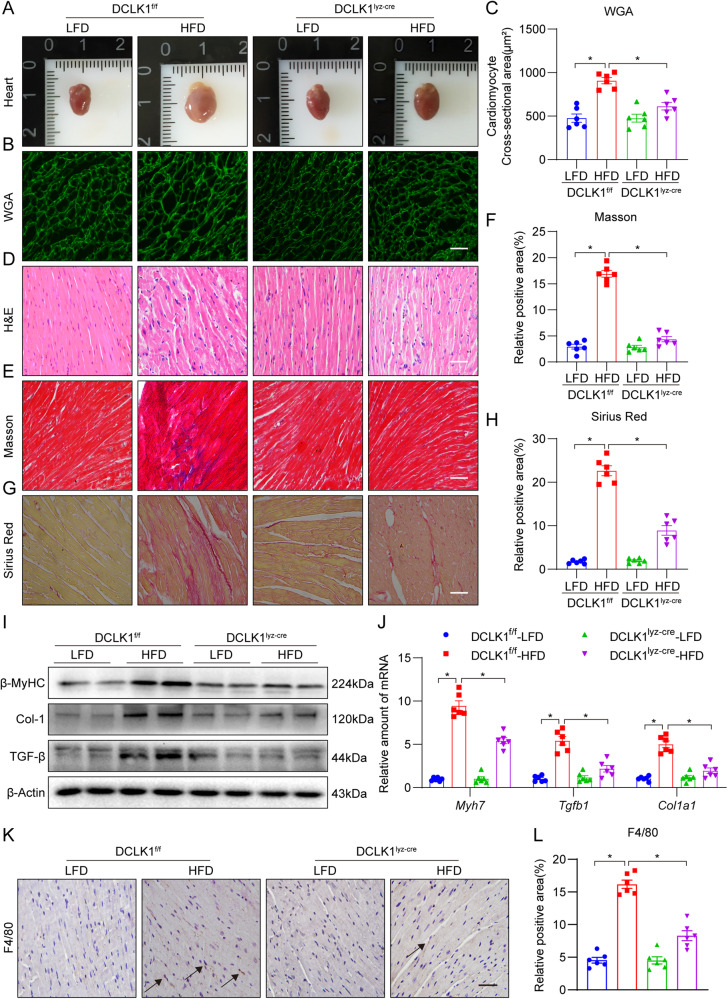
Macrophage-specific DCLK1 knockout reverses HFD-induced heart hypertrophy and fibrosis. **A** Representative fresh heart images on white background. **B**, **C** Representative WGA staining of heart tissues and quantitative analysis of myocyte area. A minimum of 100 cells were measured from different visual fields of 6 samples per group [scale bar = 50 μm; *n* = 6; one-way ANOVA followed by Tukey post hoc test; mean ± SEM; *p < 0.05]. **D** Representative hematoxylin-eosin (H&E) staining image of heart tissues [scale bar = 50 μm; *n* = 6]. **E**, **F** Representative micrographs of Masson trichrome staining and quantification of interstitial fibrotic areas (%) from Masson trichrome staining [scale bar = 50 μm; *n* = 6; one-way ANOVA followed by Tukey post hoc test; mean ± SEM; *p < 0.05]. **G**, **H** Representative micrographs of interstitial Picro-Sirius red staining and quantification of interstitial fibrotic areas (%) [scale bar = 50 μm; *n* = 6; one-way ANOVA followed by Tukey post hoc test; mean ± SEM; *p < 0.05]. **I** Representative Western blot analysis of β-MyHC, COL-1, and TGF-β in heart tissues. β-Actin was used as the loading control [*n* = 6; mean ± SEM; *p < 0.05]. **J** The mRNA levels of *Myh7*, *Col1a1*, and *Tgfb1* were detected by RT-qPCR in the heart tissues. Transcripts normalized to *Actb* [*n* = 6; one-way ANOVA followed by Tukey post hoc test; mean ± SEM; *p < 0.05]. **K**, **L** The heart was harvested to perform immunohistochemical staining for macrophage marker (F4/80) and percent quantification of F4/80-positive cells [scale bar = 50 μm; *n* = 6; one-way ANOVA followed by Tukey post hoc test; mean ± SEM; *p < 0.05].

We developed macrophage- and cardiomyocyte-specific *DCLK1* knockout mice to examine the potential role of DCLK1. Macrophage-specific *DCLK1* knockout mice (DCLK1^lyz-cre^) were developed by crossing the Lyz2-Cre mice with DCLK1 flox mice (DCLK1^f/f^). DCLK1^lyz-cre^ and DCLK1^f/f^ mice were supplemented with an LFD or an HFD for 20 weeks. DCLK1^lyz-cre^-HFD group mice showed no significant difference in their body weights from those of DCLK1^f/f^-HFD group mice (Supplementary Fig. S2A). Non-invasive echocardiography showed that macrophage-specific *DCLK1* knockout alleviated the HFD-induced cardiac dysfunction, As demonstrated by the EF and FS data in DCLK1^lyz-cre^-HFD mice (Table 1 and Supplementary Fig. S2B). Moreover, macrophage-specific *DCLK1* knockout prevented the increase in serum creatine kinase MB (CK-MB) and atrial natriuretic peptide (ANP) levels in HFD-fed mice (Table 1). Furthermore, DCLK1^lyz-cre^-HFD group mice exhibited a significant reduction in levels of serum triglycerides, total cholesterol, and low-density lipoprotein cholesterol, in comparison with DCLK1^f/f^-HFD group mice (Supplementary Fig. S2C–E). Interestingly, the decreased HDL-cholesterol level in DCLK1^f/f^-HFD mice has also been reversed by macrophage DCLK1 deletion (Supplementary Fig. S2F). Next, we examined the effect of macrophage-specific *DCLK1* knockout on cardiac morphology. Both the visual inspection of the whole heart and WGA staining of heart tissue slices revealed hypertrophic alterations in DCLK1^f/f^-HFD group mice, while macrophage-specific *DCLK1* knockout mice exhibited significantly reduced HFD-induced cardiac hypertrophy (Fig. 2A–C). Identical findings were discovered when matching the weight of the heart to the length of the tibia (Table 1). Next, H&E staining was performed to identify HFD-induced cardiac structural changes. Results showed that macrophage DCLK1 deficiency in HFD-fed DCLK1^lyz-cre^ mice reversed HFD-induced cardiac structural pathology including disorganization of myofilaments and discontinuity of some sarcomeres (Fig. 2D). The development of cardiac fibrosis is a significant contributor to the pathophysiology of heart failure brought on by an HFD. Picro-sirius red and Masson staining demonstrated that HFD-induced cardiac fibrosis was abrogated in the macrophage-specific *DCLK1* knockout mice (Fig. 2E–H). Moreover, HFD-induced cardiac fibrosis and hypertrophy were proved by an expression elevation of β-MyHC, COL-1, and TGF-β1 in the cardiac tissue of DCLK1^f/f^-HFD group mice, while this increase was not observed in DCLK1^lyz-cre^ group mice (Fig. 2I, J and Supplementary Fig. S2G). In addition, as per the immunohistochemical labeling, HFD-fed DCLK1^f/f^ mice hearts exhibited a significant elevation of immunoreactivity of a macrophage marker F4/80 in comparison with control mice, while this change was not observed in HFD-fed DCLK1^lyz-cre^ mice (Fig. 2K, L). The current findings indicate that macrophage-specific *DCLK1* knockout is quite beneficial in minimizing the hypertrophy and functioning damage of the heart.

**Table 1:** Biometric and echocardiographic measurements in experimental mice.

Variables	DCLK1^f/f^-LFD	DCLK1^f/f^-HFD	DCLK1^lyz-cre^-LFD	DCLK1^lyz-cre^-HFD
EF, %	72.25 ± 11.21	50.77 ± 10.59^*^	77.18 ± 3.07	68.92 ± 7.71^#^
FS, %	41.79 ± 9.79	25.99 ± 6.83^*^	39.81 ± 2.54	42.30 ± 8.41^#^
LVAWs, mm	1.44 ± 0.23	1.89 ± 0.37^*^	1.38 ± 0.30	1.35 ± 0.20^#^
LVAWd, mm	1.05 ± 0.15	1.33 ± 0.17^*^	0.83 ± 0.12	1.13 ± 0.13 ^ns^
LVPWs, mm	1.04 ± 0.20	1.79 ± 0.42^*^	1.08 ± 0.10	1.78 ± 0.20 ^ns^
LVPWd, mm	0.88 ± 0.15	1.39 ± 0.15^*^	0.82 ± 0.15	1.06 ± 0.26^#^
HW/TL, mg/mm	9.68 ± 0.89	14.43 ± 1.31^*^	9.78 ± 1.02	12.75 ± 0.24^#^
CK-MB, U/L	134.74 ± 35.76	211.37 ± 20.24^*^	129.78 ± 22.05	153.44 ± 17.34^#^
ANP, pg/ml	122.37 ± 26.26	221.05 ± 30.83^*^	122.97 ± 21.87	153.47 ± 23.57^#^

Transthoracic echocardiography was performed on mice at the end of the animal study. *LVPWs* LV posterior wall thickness in systole; *LVAWs* LV anterior wall thickness in systole, *LVPWd* LV posterior wall thickness in diastole, *LVAW* LV anterior wall thickness in diastole; *EF* ejection fraction, *FS* fractional shortening, *HW* heart weight, *TL* tibia length, *CK-MB* creatine kinase MB *ANP* atrial natriuretic peptide. *n* = 6 per group; *p < 0.05 compared to DCLK1^f/f^-LFD; ^#^*p* < 0.05, *ns* not significant, compare to DCLK1^f/f^-HFD. Data presented as mean ± SEM, *P* values by one-way ANOVA followed by Tukey’s post hoc test are indicated

### Cardiomyocyte-specific DCLK1 knockout did not prevent HFD-induced cardiac hypertrophy and fibrosis

**Fig. 3:**
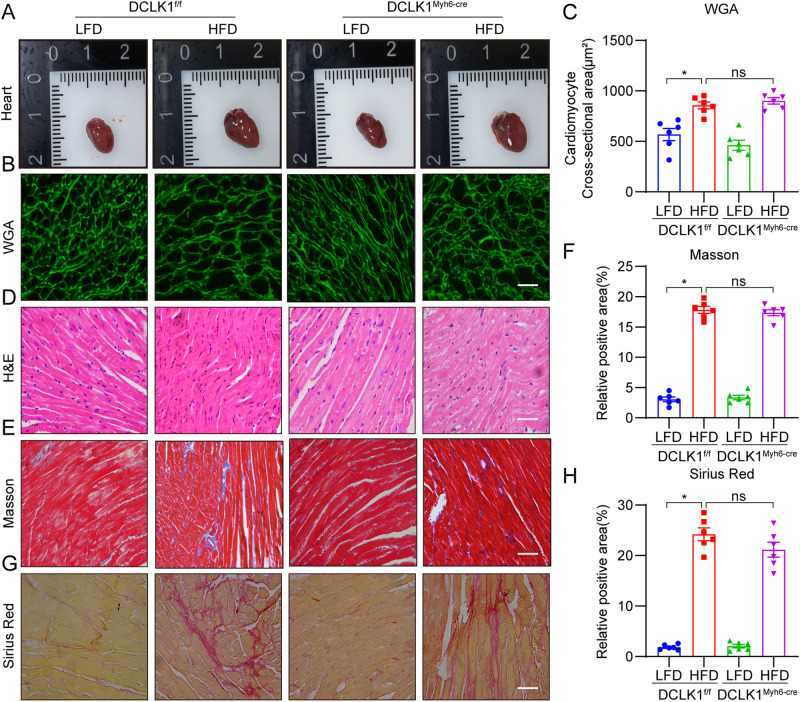
Cardiomyocyte-specific DCLK1 knockout could not reverse HFD-induced heart hypertrophy and fibrosis. **A** Representative fresh heart images on white background. **B**, **C** Representative WGA staining of heart tissues and quantitative analysis of myocyte area. A minimum of 100 cells were measured from different visual fields of 6 samples per group [scale bar = 50 μm; *n* = 6; one-way ANOVA followed by Tukey post hoc test; mean ± SEM; *p < 0.05]. **D** Representative hematoxylin-eosin (H&E) staining image of heart tissues [scale bar = 50 μm; *n* = 6]. **E**, **F** Representative micrographs of Masson trichrome staining and quantification of interstitial fibrotic areas (%) from Masson trichrome staining [scale bar = 50 μm; *n* = 6; one-way ANOVA followed by Tukey post hoc test; mean ± SEM; *p < 0.05]. **G**, **H** Representative micrographs of interstitial Picro-Sirius red staining and quantification of interstitial fibrotic areas (%) [scale bar = 50 μm; *n* = 6; one-way ANOVA followed by Tukey post hoc test; mean ± SEM; *p < 0.05].

To explore whether DCLK1 in cardiomyocytes performs a contribution to obesity-induced cardiomyopathy, cardiomyocyte-specific *DCLK1* knockout mice (DCLK1^Myh6-cre^) and DCLK1^f/f^ mice that were given LFD or HFD for 20 weeks. Results showed that the body weight of DCLK1^Myh6-cre^-HFD group mice exhibited no significant variation from that of DCLK1^f/f^-HFD group mice (Supplementary Fig. S3A). Remarkably, echocardiography that is non-invasive revealed that cardiomyocyte-specific *DCLK1* knockout failed to prevent HFD-induced cardiac dysfunction (Supplementary Fig. S3B and Table S3). Furthermore, cardiomyocyte-specific *DCLK1* knockout failed to prevent the increase in serum CK-MB and ANP values in HFD-fed mice (Supplementary Table S3). Serum lipid profile showed no beneficial effect of cardiomyocyte-specific *DCLK1* knockout in HFD-fed mice (Supplementary Fig. S3C–F). The hypertrophy alterations were visible on both a gross inspection of the complete heart and WGA labeling of the cardiac tissues not only in HFD-fed DCLK1^f/f^ mice but also in HFD-fed DCLK1^Myh6-cre^ mice, suggesting that cardiomyocyte-specific *DCLK1* knockout is unable to prevent HFD-induced cardiac hypertrophy (Fig. 3A–C). Moreover, cardiomyocyte-specific *DCLK1* knockout failed to improve HFD-induced cardiac structural changes in mice (Fig. 3D). Masson’s Trichrome and Picro-Sirius red staining also showed that HFD-fed DCLK1^Myh6-cre^ mice had collagen deposition similar to DCLK1^f/f^ mice on an HFD (Fig. 3E–H). These data indicate that cardiomyocyte-specific *DCLK1* knockout is unable to prevent HFD-induced cardiac hypertrophy and fibrosis.

### DCLK1 inhibition significantly improves HFD-induced cardiac hypertrophy and fibrosis

**Fig. 4:**
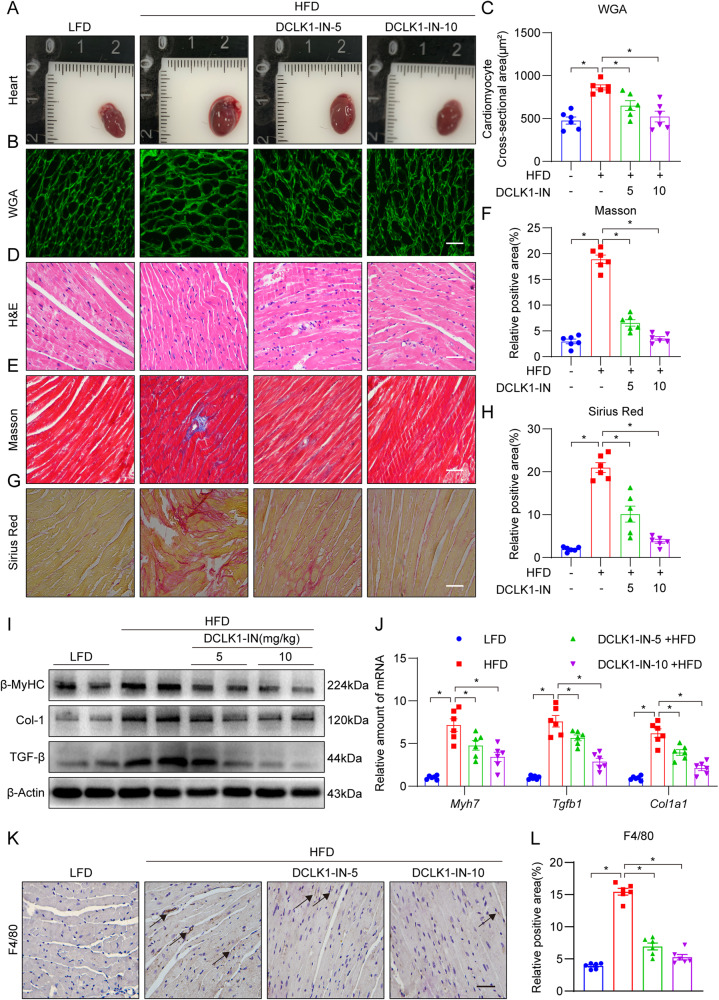
DCLK1 inhibitor significantly improved HFD-induced hypertrophy and fibrosis. **A** Representative fresh heart images on white background. **B**, **C** Representative WGA staining of heart tissues and quantitative analysis of myocyte area. A minimum of 100 cells were measured from different visual fields of 6 samples per group [scale bar = 50 μm; *n* = 6; one-way ANOVA followed by Tukey post hoc test; mean ± SEM; *p < 0.05]. **D** Representative hematoxylin-eosin (H&E) staining image of heart tissues [scale bar = 50 μm; *n* = 6]. **E**, **F** Representative micrographs of Masson trichrome staining and quantification of interstitial fibrotic areas (%) from Masson trichrome staining [scale bar = 50 μm; *n* = 6; one-way ANOVA followed by Tukey post hoc test; mean ± SEM; *p < 0.05]. **G**, **H** Representative micrographs of interstitial Picro-Sirius red staining and quantification of interstitial fibrotic areas (%) [scale bar = 50 μm; *n* = 6; one-way ANOVA followed by Tukey post hoc test; mean ± SEM; *p < 0.05]. **I** Representative Western blot analysis of β-MyHC, COL-1, and TGF-β in heart tissues. Β-Actin was used as the loading control [*n* = 6; mean ± SEM; *p < 0.05]. **J** The mRNA levels of *myh7*, *Col1a1*, and *Tgfb1* were detected by RT-qPCR in the heart tissues. Transcripts normalized to *Actb* [*n* = 6; one-way ANOVA followed by Tukey post hoc test; mean ± SEM; *p < 0.05]. **K**, **L** Mouse heart was harvested to perform immunohistochemical staining for macrophage marker (F4/80) and percent quantification of F4/80-positive cells [scale bar = 50 μm; *n* = 6; one-way ANOVA followed by Tukey post hoc test; mean ± SEM; *p < 0.05].

To strengthen our findings, we performed pharmacological inhibition of DCLK1. DCLK1-IN-1 (DCLK1-IN) is a highly selective DCLK1 inhibitor and is safe at doses up to 100 mg/kg in mice under physiological conditions [27]. Consistent with our results from *DCLK1* knockout mice, DCLK1-IN treatment (at 5 and 10 mg/kg) showed no impact on the HFD-fed mice body weights (Supplementary Fig. S4A); however, DCLK1-IN was able to ameliorate the HFD-induced cardiac dysfunction in a dose-dependent manner (Table 2 and Supplementary Fig. S4B). Moreover, therapy using DCLK1-IN was able to prevent, in a dose-dependent manner, an elevation in blood levels of CK-MB and ANP in mice that were given an HFD (Table 2). In mice fed HFD, a test of the serum lipid profile demonstrated that DCLK1-IN had a favorable impact (Supplementary Fig. S4C–F). The heart tissues of mice given an HFD were shown to have hypertrophic alterations when inspected grossly and when dyed with WGA; whereas, DCLK1-IN treatment significantly reduced the HFD-induced cardiac hypertrophy in the mice (Fig. 4A–C). Identical findings were discovered when matching the weight of the heart to the length of the tibia in mice (Table 2). Moreover, HFD-induced histological changes, such as disorganization of myofilaments, in the mouse heart were ameliorated following DCLK1-IN treatment (Fig. 4D). DCLK1-IN therapy prevented the HFD-induced cardiac fibrosis (Fig. 4E–H) and dose-dependently decreased the levels of fibrosis and hypertrophy markers β-MyHC, COL-1, and TGF-β1 in the cardiac tissue of HFD-fed mice (Fig. 4I, J, Supplementary Fig. S4G). In addition, immunohistochemical staining exhibited that hearts from the HFD-fed DCLK1^f/f^ mice showed significant elevation in F4/80 immunoreactivity in comparison with control mice, while this change was remarkably reduced in DCLK1-IN-treated HFD mice (Fig. 4K, L). These data suggest that similar to *DCLK1* knockout in macrophages, pharmacological suppression of DCLK1 prevents HFD-induced heart damage.

**Table 2:** Biometric and echocardiographic measurements in experimental mice.

Variables	Con	HFD	DCLK1-IN-5 + HFD (mg/kg)	DCLK1-IN-10 + HFD (mg/kg)
EF, %	62.50 ± 11.50	48.13 ± 5.90^*^	56.73 ± 5.67^#^	60.61 ± 3.95^#^
FS, %	33.73 ± 7.69	23.83 ± 3.51^*^	27.29 ± 3.76^#^	31.66 ± 2.45^#^
LVAWs, mm	1.92 ± 0.32	2.32 ± 0.73^*^	1.59 ± 0.27^#^	1.66 ± 0.16^#^
LVAWd, mm	1.14 ± 0.31	1.84 ± 0.71^*^	1.25 ± 0.30^#^	1.06 ± 0.20^#^
LVPWs, mm	1.37 ± 0.36	1.65 ± 0.32^*^	1.17 ± 0.11^#^	1.14 ± 0.33^#^
LVPWd, mm	1.08 ± 0.35	1.49 ± 0.25^*^	0.85 ± 0.12^#^	0.89 ± 0.34^#^
HW/TL, mg/mm	8.58 ± 0.82	10.82 ± 0.46^*^	9.41 ± 0.48^#^	8.91 ± 0.84^#^
CK-MB, U/L	140.42 ± 36.99	201.08 ± 28.93^*^	159.38 ± 29.18^#^	149.18 ± 38.64^#^
ANP, pg/ml	117.35 ± 20.59	208.43 ± 38.85^*^	155.02 ± 29.02^#^	134.08 ± 20.30^#^

Transthoracic echocardiography was performed on mice at the end of the animal study. *LVPWs* LV posterior wall thickness in systole, *LVAWs*, LV anterior wall thickness in systole, *LVPWd*, LV posterior wall thickness in diastole, *LVAW* LV anterior wall thickness in diastole, *EF* ejection fraction, *FS* fractional shortening, *HW* heart weight, *TL* tibia length, *CK-MB* creatine kinase MB, *ANP* atrial natriuretic peptide. *n* = 6 per group, *p < 0.05 compared to Con; ^#^*p* < 0.05, *ns* not significant, compare to HFD. Data presented as mean ± SEM, *P* values by one-way ANOVA followed by Tukey’s post hoc test are indicated

### Deleting DCLK1 in macrophages alleviates HFD-induced myocardial injury by inhibiting the RIP2/TAK1 signaling pathway

**Fig. 5:**
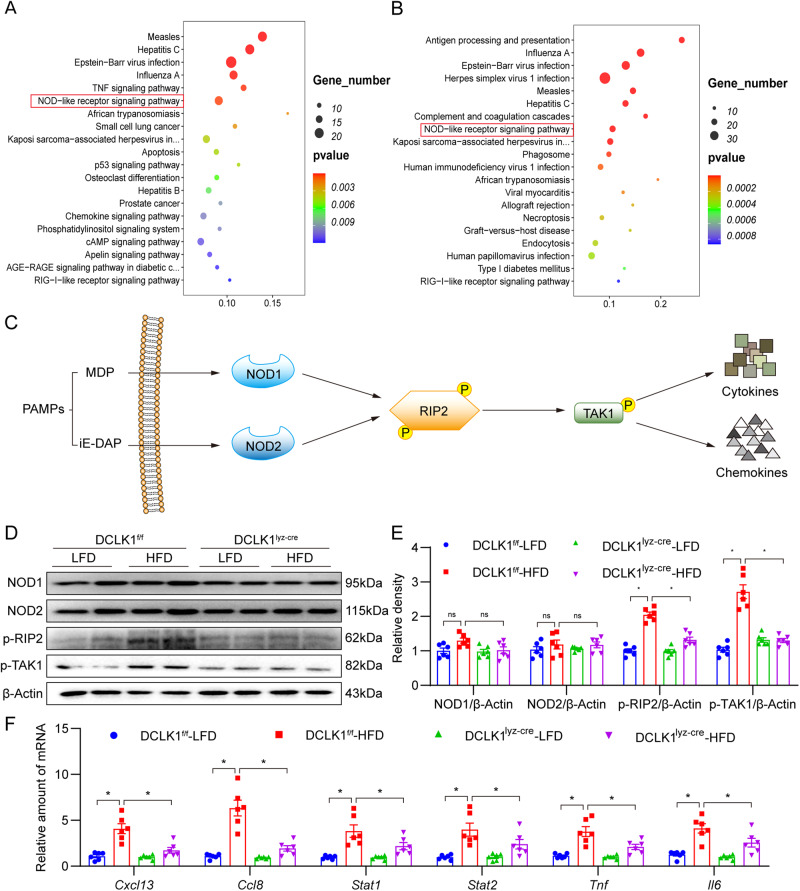
Macrophage DCLK1 knockout inhibits PA-induced RIP2/TAK1 signal pathway activation. **A**, **B** KEGG enrichment of the differential gene in HFD-treated group vs LFD-treated group (**A**) and DCLK1^lyz-cre^ + HFD-treated group vs HFD-treated group (**B**). **C** NOD-like receptor signal pathway schematic diagram. **D** Representative Western blot analysis of NOD1, NOD2, p-RIP2, and p-TAK1 in the heart tissues. Β-Actin was used as the loading control [*n* = 6; mean ± SEM; *p < 0.05]. **E** Densitometric quantification of blots in **C** [*n* = 6; one-way ANOVA followed by Tukey post hoc test; mean ± SEM; *p < 0.05]. **F** The mRNA levels of *Cxcl13*, *Ccl8*, *Stat1, Stat2, Tnf,* and *Il6* were detected by RT-qPCR in the heart tissues. Transcripts normalized to *Actb* [*n* = 6; one-way ANOVA followed by Tukey post hoc test; mean ± SEM; *p < 0.05].

To understand how macrophage-specific DCLK1 regulates obesity-induced cardiomyopathy, we carried RNA sequencing of the cardiac tissues from various mice groups. Results showed that 758 genes were differentially regulated (412 upregulated and 346 downregulated) when comparing DCLK1^f/f^-HFD and DCLK1^f/f^-LFD groups (Supplementary Fig. S5A, B), while 600 differentially regulated genes were identified (234 upregulated and 366 downregulated) when comparing DCLK1^lyz-cre^-HFD and DCLK1^f/f^-HFD groups (Supplementary Fig. S5C). KEGG enrichment analysis of these differentially regulated genes revealed the presence of NOD-like receptor signaling pathway among the ten leading pathways in both sets of analyses (Fig. 5A, B), which is an indication of its participation in cardioprotection following macrophage-specific *DCLK1* deletion.

The NOD-like receptor is a pathogen recognition receptor that has evolved as an important controller of the inflammatory process [28]. Additionally, the NOD-like receptor is a possible treatment option for a wide variety of inflammatory and autoimmune disorders [29, 30]. In response to being stimulated by pathogen-associated molecular patterns or damage-associated molecular patterns, NOD1 and NOD2 stimulate the activation of receptor-interacting protein kinase 2 (RIPK2), which phosphorylates transforming growth factor-β activated kinase 1 (TAK1) to activate nuclear factor kappa-B (NF-κB) pathway, which eventually results in the synthesis of pro-inflammatory factors [31–33] (Fig. 5C).

Therefore, we investigated whether DCLK1 regulates key proteins of the NOD-like receptor signaling pathway. Interestingly, we found that macrophage-specific *DCLK1* deletion did not affect the protein levels of NOD1/2 but significantly decreased the HFD-induced RIP2 and TAK1 phosphorylation in the cardiac tissue (Fig. 5D, E). In addition, immunofluorescence staining showed that p-RIP2 is co-localized with the F4/80-positive macrophages in mouse hearts (Supplementary Fig. S6A). HFD increased the p-RIP2 level in cardiac macrophages, while this change was reversed in DCLK1^lyz-cre^-HFD mice (Supplementary Fig. S6A). Identical findings were detected in HFD-fed mice treated with the DCLK1 inhibitor DCLK1-IN (Supplementary Fig. S6B, C). Furthermore, we examined the expression of six target genes that are controlled by the NOD-like receptor signaling pathway during inflammatory response by real-time qPCR [34, 35]. Results revealed that HFD raises these six genes expression in the mouse heart, while macrophage-specific *DCLK1* knockout reversed these changes (Fig. 5F). Pharmacological inhibition of DCLK1 also showed a similar effect (Supplementary Fig. S6D). These results indicate that macrophage-specific *DCLK1* knockout or DCLK1 inhibition alleviates HFD-induced myocardial injury by inhibiting the RIP2/TAK1 signaling pathway.

### Macrophage-specific DCLK1 deletion inhibits PA-induced activation of the RIP2/TAK1 signaling pathway and inflammatory responses

**Fig. 6:**
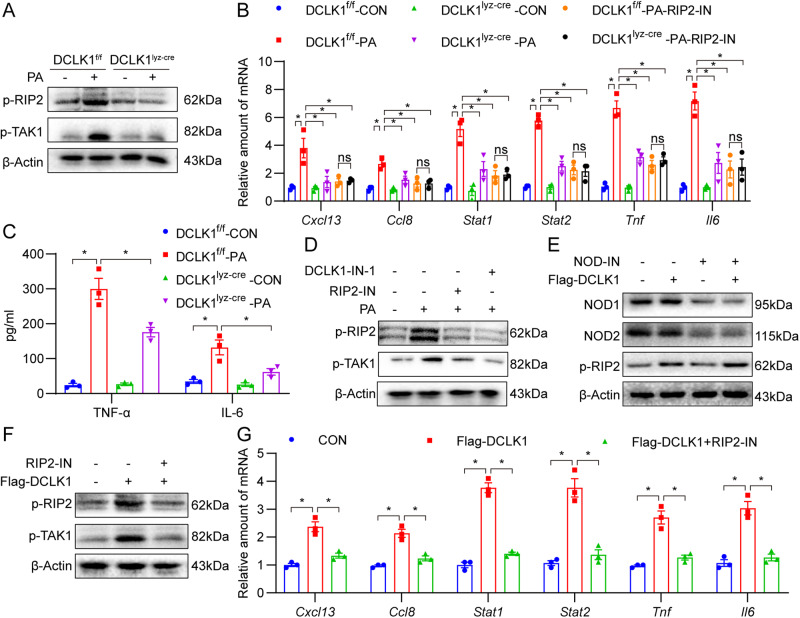
Macrophage DCLK1 knockout inhibits PA-induced RIP2/TAK1 signal pathway activation. **A** MPMs were exposed to 200 μM PA or 10% BSA for 30 min. Cell lysates were probed for p-RIP2 and p-TAK1 to assess pathway activation. β-actin was used as loading control. [*n* = 3; mean ± SEM; *p < 0.05]. **B** MPMs from DCLK1^f/f^ or DCLK1^lyz-cre^ mice were exposed to 200 μM PA for 12 h, pre-treated with or without 5 μM RIP2-IN for 1 h. mRNA levels of *Cxcl13, Ccl8, Stat1, Stat2, Tnf,* and *Il6* in cells were examined by RT-qPCR assay. Transcripts normalized to *Actb* [*n* = 3; one-way ANOVA followed by Tukey post hoc test; mean ± SEM; *p < 0.05]. **C** MPMs were exposed to 200 μM PA or 10% BSA (con) for 12 h. Levels of TNF-α and IL-6 protein levels in culture media were determined [*n* = 3; one-way ANOVA followed by Tukey post hoc test; mean ± SEM; *p < 0.05]. **D** MPMs were pre-treated with 10 μM DCLK1-IN or 5 μM RIP2-IN for 1 h and then exposed to 200 μM PA or 10% BSA for 30 min. Representative western blot analysis of p-RIP2 and p-TAK1 in the MPMs. β-actin was used as the loading control [*n* = 3; mean ± SEM; *p < 0.05]. **E** MPMs transfected with Flag-DCLK1 were treated with or without 5 μM NOD-IN for 12 h. Representative Western blot analysis of NOD1, NOD2, and p-RIP2 in the MPMs. β-actin was used as the loading control [*n* = 3; mean ± SEM; *p < 0.05]. **F**, **G** MPMs transfected with Flag-DCLK1 were treated with or without 5 μM RIP2-IN. Representative western blot analysis of p-RIP2 and p-TAK1 in MPMs (**F**). β-actin was used as the loading control [*n* = 3; mean ± SEM; *p < 0.05]. RT-qPCR showed the mRNA levels of *Cxcl13, Ccl8, Stat1, Stat2, Tnf,* and *Il6* in MPMs. Transcripts normalized to *Actb* (**G**) [*n* = 3; one-way ANOVA followed by Tukey post hoc test; mean ± SEM; *p < 0.05].

We further explored the underlying mechanism using in vitro cultured macrophages challenged with palmitate (PA), a saturated fatty acid implicated in obesity-associated cardiomyopathy [36]. Obesity-induced cardiomyopathy is characterized by inflammation due to elevated levels of free fatty acids, such as PA. We recovered MPMs from DCLK1^lyz-cre^ and DCLK1^f/f^ mice and subjected them to PA. Interestingly, western blotting confirmed that p-RIP2 and p-TAK1 levels were increased in PA-challenged MPMs from DCLK1^f/f^ mice, while macrophage-specific DCLK1 deficiency prevented this increase (Fig. 6A and Supplementary Fig. S7A). Also, PA-induced mRNA levels of the six target genes, which have been reported to be regulated by the NOD-like receptor signaling pathway [34, 35], were abrogated in DCLK1-deficient MPMs (Fig. 6B). When we used RIP2-IN to block RIP2 activation in DCLK1^−/−^ macrophages, DCLK1 deletion failed to further reduce PA-induced inflammatory gene expression in macrophages with RIP2 inhibition (Fig. 6B). Moreover, *DCLK1* deletion prevented the PA-induced TNF-α and IL-6 production in MPMs (Fig. 6C). Pharmacological inhibition of DCLK1 by DCLK1-IN also had similar effects (Supplementary Fig. S7C, D). PA-induced RIP2/TAK1 phosphorylation in MPMs was significantly inhibited by DCLK1-IN and the RIP2 inhibitor RIP2-IN (Fig. 6D and Supplementary Fig. S7E).

We then transfected MPMs with Flag-DCLK1 to determine whether DCLK1 overexpression affects RIP2/TAK1 signal. DCLK1 overexpression increases the levels of p-RIP2 and p-TAK1, but not NOD1/2 (Fig. 6E, F and Supplementary Fig. S7F, G). NOD1/2 inhibition using NOD-IN reduced the level of NOD1/2 but failed to inhibit Flag-DCLK1-induced RIP2 phosphorylation (Fig. 6E and Supplementary Fig. S7F), confirming that DCLK1 regulates RIP2 activation in a NOD1/2-independent manner. As expected, the RIP2 inhibitor RIP2-IN significantly blocked the Flag-DCLK1-induced RIP2/TAK1 phosphorylation (Fig. 6F and Supplementary Fig. S7G) and transcription of the six target genes (Fig. 6G). In contrast, RIP2 overexpression had no effect on DCLK1 levels in MPMs (Supplementary Fig. S7H, I). These results indicate that DCLK1 promotes inflammatory responses through RIP2 activation and that DCLK1 activates the NOD1/2-independent RIP2/TAK1 pro-inflammatory pathway in PA-challenged MPMs.

### DCLK1-dependent macrophage factors promote cardiomyocyte hypertrophy and cardiac fibroblast fibrogenesis

**Fig. 7:**
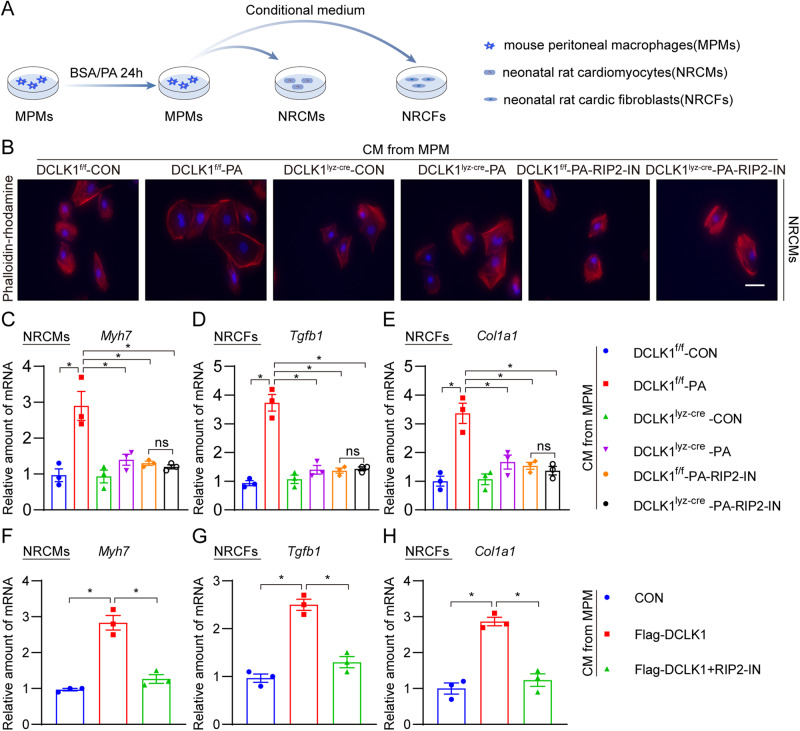
DCLK1-dependent macrophage factors enhance heart hypertrophy and fibrogenesis. **A** Schematic showing the experimental setup to assess the effect of macrophage-derived factors on NRCMs and NRCFs. MPMs harvested from DCLK1^f/f^ and DCLK1^lyz-cre^ mice were pre-treated with or without 5 μM RIP2-IN for 1 h and then exposed to 200 μM PA for 24 h. BSA was used as a control. Conditioned media (CM) was collected and applied to NRCMs or NRCFs in a 1:1 ratio with fresh media. Cells were analyzed after 24 h. **B** TRITC phalloidin (red) staining of NRCMs showing the effect of DCLK1 knockout in macrophages on PA-induced hypertrophic responses. Slides were counterstained with DAPI (blue). Quantification of cell size was shown in the supplementary file [scale bar = 50 μm]. **C**–**E** NRCMs or NRCFs were challenged with the CM of MPMs as indicated. mRNA levels of *Myh7*, *Col1a1*, and *Tgfb1* were determined by RT-qPCR. Transcripts were normalized to *Actb* [*n* = 3; one-way ANOVA followed by Tukey post hoc test; mean ± SEM; *p < 0.05]. **F**–**H** mRNA levels of *myh7*, *Col1a1*, and *Tgfb1*. Transcripts were normalized to *Actb* [*n* = 3; one-way ANOVA followed by Tukey post hoc test; mean ± SEM; *p < 0.05].

Considering that the cellular crosstalk between macrophages and heart cells promotes obesity-related cardiomyopathy [37], we evaluated the impacts of macrophage-secretory factors on cardiomyocytes and cardiac fibroblasts. MPMs were exposed to PA for 24 h and CM was retrieved and then incubated with NRCMs and neonatal rat cardiac fibroblasts (NRCFs) (Fig. 7A). CM from PA-challenged DCLK1^f/f^ MPMs increased the hypertrophy and *Myh7* gene transcription in cultured NRCMs, while CM from PA-challenged DCLK1^lyz-cre^ MPMs failed to induce these changes in NRCMs (Fig. 7B, C and Supplementary Fig. S8). Moreover, CM from PA-challenged DCLK1^f/f^ MPMs increased the mRNA levels of *Tgfb1* and *Col1a1* in NRCFs, while CM from DCLK1-deficient MPMs had no pro-fibrotic effects on NRCFs (Fig. 7D, E). As expected, RIP2 inhibition by RIP2-IN in macrophages also normalized the protective effects of DCLK1 deletion against macrophage factors-induced cardiomyocyte hypertrophy and cardiac fibroblast fibrogenesis (Fig. 7B–E and Supplementary Fig. S8). To further confirm the role of macrophage-specific DCLK1, we transfected MPMs with the Flag-DCLK1 plasmid. As expected, CM from DCLK1-overexpressing MPMs increased the mRNA levels of *Myh7* in NRCMs, and *Tgfb1* and *Col1a1* in NRCFs, while these changes were alleviated upon RIP2-IN treatment of MPMs (Fig. 7F–H). These data indicate that DCLK1-dependent inflammatory factors produced by macrophages promote hypertrophy and fibrosis in cardiac cells.

## Discussion

The present research evaluated the impact of macrophage-specific DCLK1 in the establishment of obesity-associated cardiomyopathy. We observed that DCLK1 level is elevated in the cardiac tissue of HFD-fed mice. Myeloid cell-specific *DCLK1* deletion or pharmacological inhibition of DCLK1 reduced cardiac hypertrophy and fibrosis, thereafter, restored heart function in HFD-fed mice. RNA sequencing analysis showed that DCLK1 deficiency exerted cardioprotective effects by suppressing RIP2/TAK1 activation and inflammatory responses of macrophages. Upon HFD/PA challenge, DCLK1 mediates RIP2/TAK1 phosphorylation and subsequent inflammation, which is independent of NOD1/2. Our study provides extensive proof that macrophage-specific DCLK1 has a deleterious impact on obesity-associated cardiomyopathy by inducing RIP2/TAK1 activation and inflammatory responses in macrophages, which subsequently promotes cardiac hypertrophy and fibrosis. These key findings are summarized in the graphical abstract.

Targeting macrophage activation and macrophage-mediated inflammation can offer novel strategies for the management of obesity-related cardiomyopathy. Latest research has demonstrated that DCLK1 acts a key function in a variety of illnesses, such as colitis [20, 38–40]. Through controlling mucosal immune reactions, the Notch-DCLK1 axis is vital for the establishment of murine and human colitis by modulating mucosal immune reactions [38]. DCLK1 controls colonic inflammatory reaction and colonic epithelial integrity [40]. Limited proof exists about the involvement of DCLK1 in macrophages. This research reports that macrophage-specific DCLK1 performs a key part in obesity-related inflammation and cardiomyopathy. Interestingly, we found that macrophage- but not cardiomyocyte-specific *DCLK1* knockout prevented HFD-induced cardiac hypertrophy and fibrosis. Recent studies have indicated that macrophage activation and inflammation play essential roles in lipid metabolism and insulin resistance [41–45]. We also observed that both macrophage-specific *DCLK1* knockout and pharmacological inhibition of DCLK1 have beneficial effects in HFD-fed mice. Although we do not yet know whether DCLK1-IN has any effect on cardiac function in healthy hearts, we found that it plays a role in obese cardiomyopathy. Although our study focused on the heart, our data also showed that DCLK1 deletion in macrophages or pharmacological inhibition significantly reversed hyperlipidemia profile in HFD-fed mice, suggesting that DCLK1-mediated macrophage activation plays a role in lipid metabolism. In addition, macrophages originate from blood monocytes that leave the circulation to differentiate in different tissues upon obesity. Thus, macrophage-specific DCLK1 deficiency may also reverse HFD-induced inflammatory injuries in other tissues. The potential involvements of DCLK1 in obesity and other obesity-induced diseases are interesting and deserve further research.

NOD1 and NOD2 mediate innate immunity induced by bacterial peptidoglycan derivatives [31]. Moreover, the latest investigations have revealed that NOD1/RIP2 signaling complex regulates remodeling, inflammatory reaction as well as mitochondrial energy metabolism in stressed cardiomyocytes [46]. NOD2 is also a critical component of the signal transduction pathway that promotes cardiac injury by exacerbating inflammation via MMP-9 activation [47]. RIP2-deficiency ameliorates cardiac hypertrophy, inflammation, and fibrosis by controlling several signaling pathways [46]. Also, the inhibition of downstream targets of RIP2 includes TAK1. TAK1 can be inhibited by tabersonine and attenuates the angiotensin II-induced cardiac remodeling and dysfunction [48]. Herein, we demonstrated that the RIP2-TAK1 signaling pathway in macrophages is activated by DCLK1 during obesity, and this activation is independent of NOD1/2. Our data also show that DCLK1-RIP2/TAK1 axle in macrophages also regulates the expression of chemokines (*Ccl8* and *Cxcl13*), which further promote cardiac macrophage infiltration [49, 50]. Decreased macrophage population also contributes greatly to the cardioprotective effects of DCLK1 blockage. Together, DCLK1-mediated RIP2-TAK1 activation in macrophages leads to inflammatory cytokine production and cardiac macrophage infiltration, promoting myocardial injury in obese conditions.

DCLK1 has a crucial function in the control of carcinogenesis and metastasis through the NOTCH, WNT, RTK, TGF, and Hedgehog signaling pathways [51]. Through controlling the Wnt/-catenin pathway, DCLK1 aids in the maintenance of tumor stem cells [52]. Our study is the first showing that the DCLK1-RIP2-TAK1 axis is regulated by DCLK1, which is different from the other known DCLK1 downstream pathways. In terms of NOD1 and NOD2 activation [53], the signaling pathways controlled through RIPK2 have been thoroughly explored. Oligomeric NOD complexes recruit RIP2 by interacting with CARD-CARD [54]. RIP2 works as a signaling molecule downstream of NOD1, NOD2, and toll-like receptors [53], and it causes gut inflammation independent of NOD2. Watanabe et al. revealed that NOD1/2-independent RIP2 activation has a fundamental contribution in both murine colitis and human inflammatory bowel disease [55]. Interestingly, we found that DCLK1 regulates the RIP2-TAK1 signaling pathway independent of NOD1/2. Our results indicated that DCLK1 gene knockout and pharmacological inhibition are similarly effective in reducing heart injury in mice. Therefore, it is possible that DCLK1 acts as a regulator of downstream RIP2/TAK1 inflammatory signaling through its kinase phosphorylation function. However, a limitation of this study is that we do not know how DCLK1 promotes RIP2 phosphorylation. In a preliminary study, we showed no interaction between DCLK1 and RIP2 in PA-challenged macrophages (Supplementary Fig. S9), indicating that DCLK1 activates RIP2 via middle mediators. So far, no kinases have been reported to directly phosphorylate RIP2. Therefore, it will be very interesting to explore the mediating kinases between DCLK1 and RIP2 in the future.

Another limitation of this study is the unclear mechanism by which HFD/PA induces or activates DCLK1 in macrophages. We observed that HFD induces the expression of DCLK1 in the heart which is particularly important since there are no known physiological activators of DCLK1. In cancer, potential mutations in the autoinhibitory domain of DCLK1 might increase its kinase activity. In the nervous system, where DCLK1 was first identified, a neuronal calcium sensor family protein, HPCAL1, acts as a potential activator of DCLK1 [56]. Besides having calcium-regulatory roles, HPCAL1 is strongly associated with myocardial infarction and coronary heart disease [57]. It would be interesting to investigate whether cardiomyocyte-specific HPCAL1 activates DCLK1 in infiltrated or circulating macrophages during obese conditions.

In conclusion, we identified a relationship between macrophage DCLK1 and cardiomyopathy caused by obesity. By controlling the RIP2/TAK1 signaling pathway and macrophage activation, we discovered that DCLK1 has a detrimental impact on obesity-induced cardiomyopathy. Inhibition of DCLK1 with pharmacological agents significantly mitigates cardiomyopathy in mice fed an HFD. Targeting DCLK1 may therefore be a feasible therapy or prophylactic approach for the control of cardiomyopathy caused by obesity.

## Supplementary information

Revised Suppl file

aj-checklist

## Data Availability

All data supporting the findings of this study are available from the corresponding authors upon reasonable request.
