# Supplementary material for: Macrophage DCLK1 promotes obesity-induced cardiomyopathy via activating RIP2/TAK1 signaling pathway
Source: Cell Death Dis. 2023 Jul 13;14(7):419. doi: 10.1038/s41419-023-05960-4 (PMC10345119; doi:10.1038/s41419-023-05960-4)
Supplement: Supplementary file 1 — Revised Suppl file [file 41419_2023_5960_MOESM1_ESM.docx]

***Supporting Information***

**Macrophage DCLK1 promotes obesity-induced cardiomyopathy via activating RIP2/TAK1 signaling pathway**

**Supplementary Table S1.** Resources of key reagents used in this study.

| REAGENT/ RESOURCES | SOURCE | CATALOGUE |
| --- | --- | --- |
| ***Antibodies*** |  |  |
| α-actin | Abcam | ab9465 |
| Vimentin | Abcam | ab8978 |
| CD68 | Abcam | ab955 |
| F4/80 | Cell Signaling Technology | 30325 |
| Alexa-488 conjugated Goat Anti Mouse (IgG) secondary antibody | Abcam | ab150117 |
| Alexa-594 conjugated Goat Anti Rabbit (IgG) secondary antibody | Abcam | ab150080 |
| Rabbit IgG | Proteintech | B900610 |
| β-MyHC | Abcam | ab50967 |
| Collagen Ⅰ | Abcam | ab34710 |
| DCLK1 | Cell Signaling Technology | 62257 |
| TGF-β | Abcam | ab92486 |
| NOD1 | Cell Signaling Technology | 3545 |
| NOD2 | Abcam | Ab31488 |
| p-RIP2 | Affinity | AF0049 |
| p-TAK1 | Cell Signaling Technology | 4537 |
| β-actin | Santa Cruz Biotechnology | sc-47778 |
| ***Chemicals, Peptides, and Recombinant Proteins*** |  |  |
| DCLK1-IN-1 (DCLK1-IN) | Bidepharm | BD01203503 |
| PA | SIGMA | P9767-5G |
| PEI | Polysciences | 23966-1 |
| RPMI-1640 | Invitrogen | 11875093 |
| DMEM | Invitrogen | 11965092 |
| Opti MEM | Invitrogen | 31985070 |
| FBS | Invitrogen | 16140071 |
| beef extract | Solarbio | G8270 |
| tryptone | OXOID | LP0042B |
| sodium chloride | Solarbio | S8210 |
| soluble starch | Solarbio | G8300 |
| RNAiso Plus | Takara | 9109 |
| RIPA buffer | Beyotime Biotech | P0013B |
| Tween 20 | Aladdin | T104863 |
| non-fat milk | B&D | 6307915 |
| TRIzol Reagent | Invitrogen | 15596026 |
| Bovine serum albumin | Sigma-Aldrich | A1933 |
| DAPI | Beyotime Biotech | C1006 |
| Protein Phosphatase Inhibitor | Solarbio | P1260 |
| PMSF | Sangon Biotech | A100754 |
| protease inhibitor cocktail | Merck | 539133 |
| HEPES | Invitrogen | 15630080 |
| EGTA | Sigma | 324626 |
| MgCl_2_ | Sigma | 68475 |
| MnCl_2_ | Sigma | M1787 |
| 5x SDS loading buffer | Beyotime Biotech | P0015L |
| glycerol | Sigma | G5516 |
| KCl | Sigma | P9541 |
| CMC-Na | Sigma | 419311 |
| ***Staining reagents and kit*** |  |  |
| Pierce ECL Western Blotting Substrate | Thermo Fisher | 32106 |
| H&E Staining kit | Solarbio | G1120 |
| PrimeScript RT with gDNA Eraser | Takara | RR047A |
| iQ SYBR Green Supermix | Bio-Rad | 1708882 |
| Mouse TNF-α ELISA kit | Invitrogen | 88-7324-77 |
| Mouse IL-6 ELISA | Invitrogen | 88-7064-88 |
| Atrial Natriuretic Peptide (ANP) determination kit | Nanjing Jiancheng Bioengineering Institute | H180 |
| Creatine Kinase MB Isoenzyme (CK-MB) determination kit | Nanjing Jiancheng Bioengineering Institute | E006–1–1 |
| Total cholesterol (TCH) | Nanjing Jiancheng Bioengineering Institute | A111-1-1 |
| Triglycerides (TG) | Nanjing Jiancheng Bioengineering Institute | A110-1-1 |
| Low-density lipoprotein-cholesterol (LDL-C) | Nanjing Jiancheng Bioengineering Institute | A113-1-1 |
| High-density lipoprotein-cholesterol (HDL-C) | Nanjing Jiancheng Bioengineering Institute | A112-1-1 |
| FITC-conjugated wheat-germ agglutinin | Gene Tex | GTX01502 |
| Rhodamine phalloidin | Solarbio | CA1610–300 T |
| Sirius Red Staining kit | Solarbio | S8060 |
| Masson’s Trichrome kit | Solarbio | G1340 |
| **Experimental Models: Organisms/Strains** |  |  |
| C57BL/6JGpt | GemPharmatech Co.,Ltd | Strain NO. N000013 |
| B6/JGpt-Dclk1em1Cflox/Gpt | GemPharmatech Co.,Ltd | Strain NO. T014962 |
| B6/JGpt-Lyz2em1Cin(iCre)/Gpt | GemPharmatech Co.,Ltd | Strain NO. T003822 |
| B6/JGpt-H11em1Cin(Myh6iCre)/Gpt | GemPharmatech Co.,Ltd | Strain NO. T004713 |
| ***Oligonucleotides*** |  |  |
| Real-time PCR primers used in this study | Thermo Fisher | See Table S4 |
| ***Plasmid*** |  |  |
| Flag-DCLK1 | MiaolingBio | P0562 |
| Flag-RIP2 | Suzhou Jinweizhi Biotechnology Co., LTD | N/A |
| ***Software and Algorithms*** |  |  |
| ImageJ (imagej.nih.gov/ij/) | NIH | 1.8.0_172 |
| Prism 8 (www.graphpad.com) | GraphPad | Prism 9.0.1 |
| ***Other*** |  |  |
| NanoDrop2000 | Thermo Scientific | N/A |

**Supplementary Table S2.** Primer sequences for real-time qPCR assay in this study

.

| **Gene** | **Species** | **Forward Primer (5’-3’)** | **Reverse Primer (5’-3’)** |
| --- | --- | --- | --- |
| *Il6* | Mouse | GAGGATACCACTCCCAACAGACC | AAGTGCATCATCGTTGTTCATACA |
| *Tnf* | Mouse | TGATCCGCGACGTGGAA | ACCGCCTGGAGTTCTGGAA |
| *Actb* | Mouse | CCGTGAAAAGATGACCCAGA | TACGACCAGAGGCATACAG |
| *Tgfb1* | Mouse | CTCCCGTGGCTTCTAGTGC | GCCTTAGTTTGGACAGGATCTG |
| *Col1a1* | Mouse | AATGGTGCTCCTGGTATTGC | GGTCCTCGTTTTCCTTCTT |
| *Cxcl13* | Mouse | GGCCACGGTATTCTGGAAGC | GGGCGTAACTTGAATCCGATCTA |
| *Ccl8* | Mouse | TCTACGCAGTGCTTCTTTGCC | AAGGGGGATCTTCAGCTTTAGTA |
| *Stat1* | Mouse | TCACAGTGGTTCGAGCTTCAG | GCAAACGAGACATCATAGGCA |
| *Stat2* | Mouse | TCCTGCCAATGGACGTTCG | GTCCCACTGGTTCAGTTGGT |
| *Myh7* | Mouse | GCCCAGTACCTCCGAAAGTC | GCCTTAACATACTCCTCCTTGTC |
|  |  |  |  |

**Supplementary Table S3.** Biometric and Echocardiographic Measurements in Experimental Mice.

| Variables | DCLK1^f/f^-LFD | DCLK1^f/f^-HFD | DCLK1^Myh6-cre^-LFD | DCLK1^Myh6-cre^-HFD |
| --- | --- | --- | --- | --- |
| EF, % | 81.40±3.84 | 75.05±2.79^*^ | 80.46±4.26 | 77.34±3.00 ^ns^ |
| FS, % | 44.01±4.08 | 38.37±1.97^*^ | 41.30±5.67 | 38.80±2.45 ^ns^ |
| LVAWs, mm | 0.81±0.35 | 1.04±0.74^*^ | 0.85±0.26 | 0.96±0.65 ^ns^ |
| LVAWd, mm | 1.36±0.21 | 1.63±0.13^*^ | 1.45±0.15 | 1.41±0.51^ns^ |
| LVPWs, mm | 1.14±0.23 | 1.38±0.31^*^ | 1.08±0.11 | 1.28±0.40 ^ns^ |
| LVPWd, mm | 0.68±0.35 | 1.99±0.25^*^ | 0.72±0.15 | 1.86±0.56 ^ns^ |
| HW/TL, mg/mm | 7.51±0.55 | 10.33±0.84^*^ | 7.12±0.71 | 9.71±0.66 ^ns^ |
| CK-MB, U/L | 134.03±38.77 | 226.67±39.61^*^ | 131.17±23.99 | 206.10±48.35 ^ns^ |
| ANP, pg/ml | 132.15±32.33 | 255.95±38.75^*^ | 145.53±39.61 | 256.40±50.06 ^ns^ |

Transthoracic echocardiography was performed on mice at the ending of the animal study. LVPWs, LV posterior wall thickness in systole; LVAWs, LV anterior wall thickness in systole; LVPWd, LV posterior wall thickness in diastole; LVAW, LV anterior wall thickness in diastole; EF, ejection fraction; FS, fractional shortening; HW, heart weight; TL, tibia length; CK-MB, Creatine kinase MB; ANP, atrial natriuretic peptide. n=6 per group; *p < 0.05 compared to DCLK1^f/f^-LFD; ns = not significant, compare to DCLK1^f/f^-HFD. Data presented as mean ± SEM, P-values by one-way ANOVA followed by Tukey’s post hoc test are indicated.


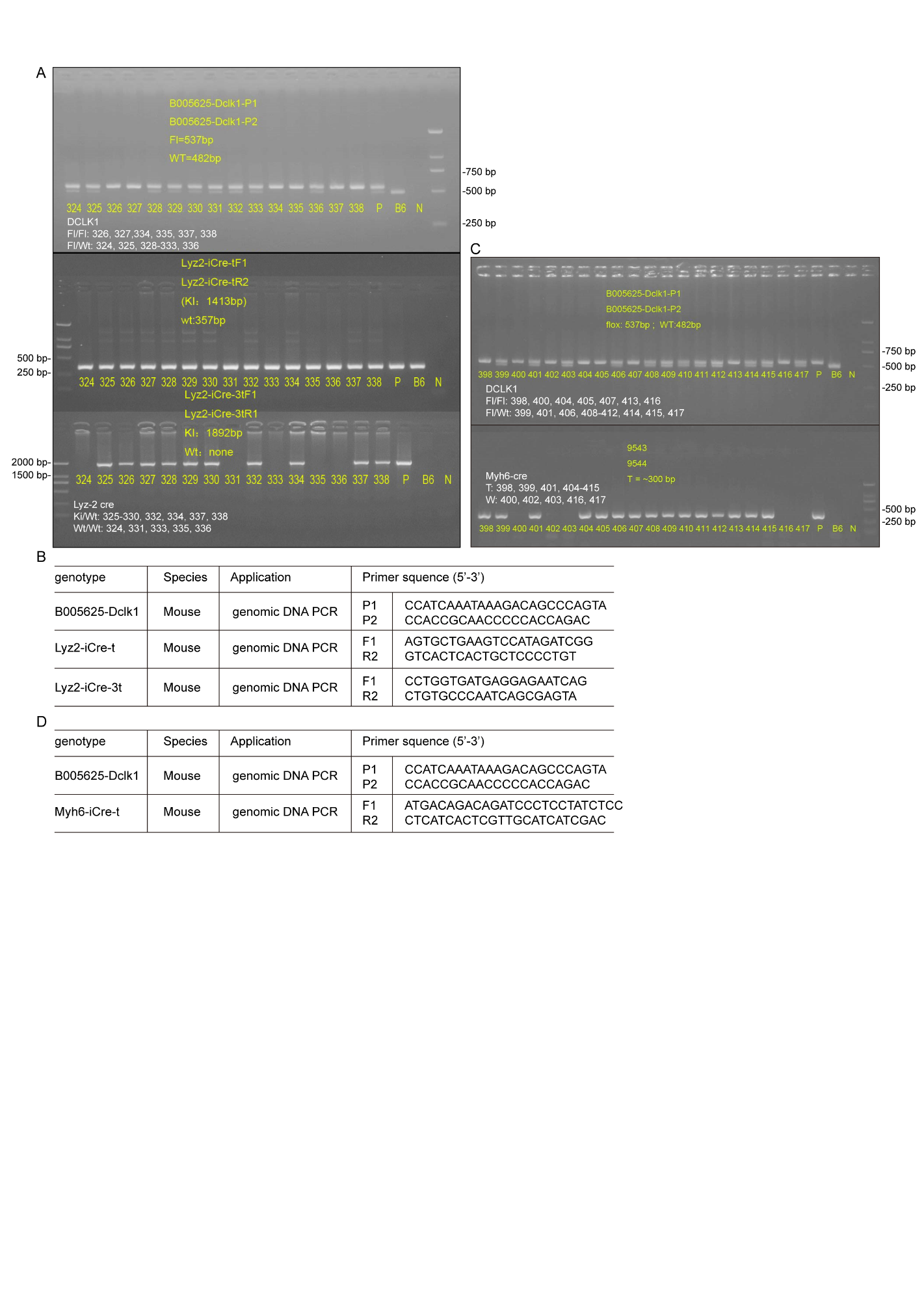


**Supplementary Figure S1: The genotyping data of macrophage-specific DCLK1 knockout mice and cardiomyocyte-specific DCLK1 knockout mice.**

(A) Genotyping was performed using genomic DNA isolated from tail clip samples. PCR products were separated using agarose gel electrophoresis to identify products of 537 bp (DCLK1f/f), and 1892 bp (DCLK1lyz-cre). (B) The primers used to genotype analysis of macrophage-specific DCLK1 knockout mice. (C)Genotyping was performed using genomic DNA isolated from tail clip samples. PCR products were separated using agarose gel electrophoresis to identify products of 537 bp (DCLK1f/f), and ~300 bp (DCLK1Myh6-cre). (D) The primers used to genotype analysis of cardiomyocyte-specific DCLK1 knockout mice.


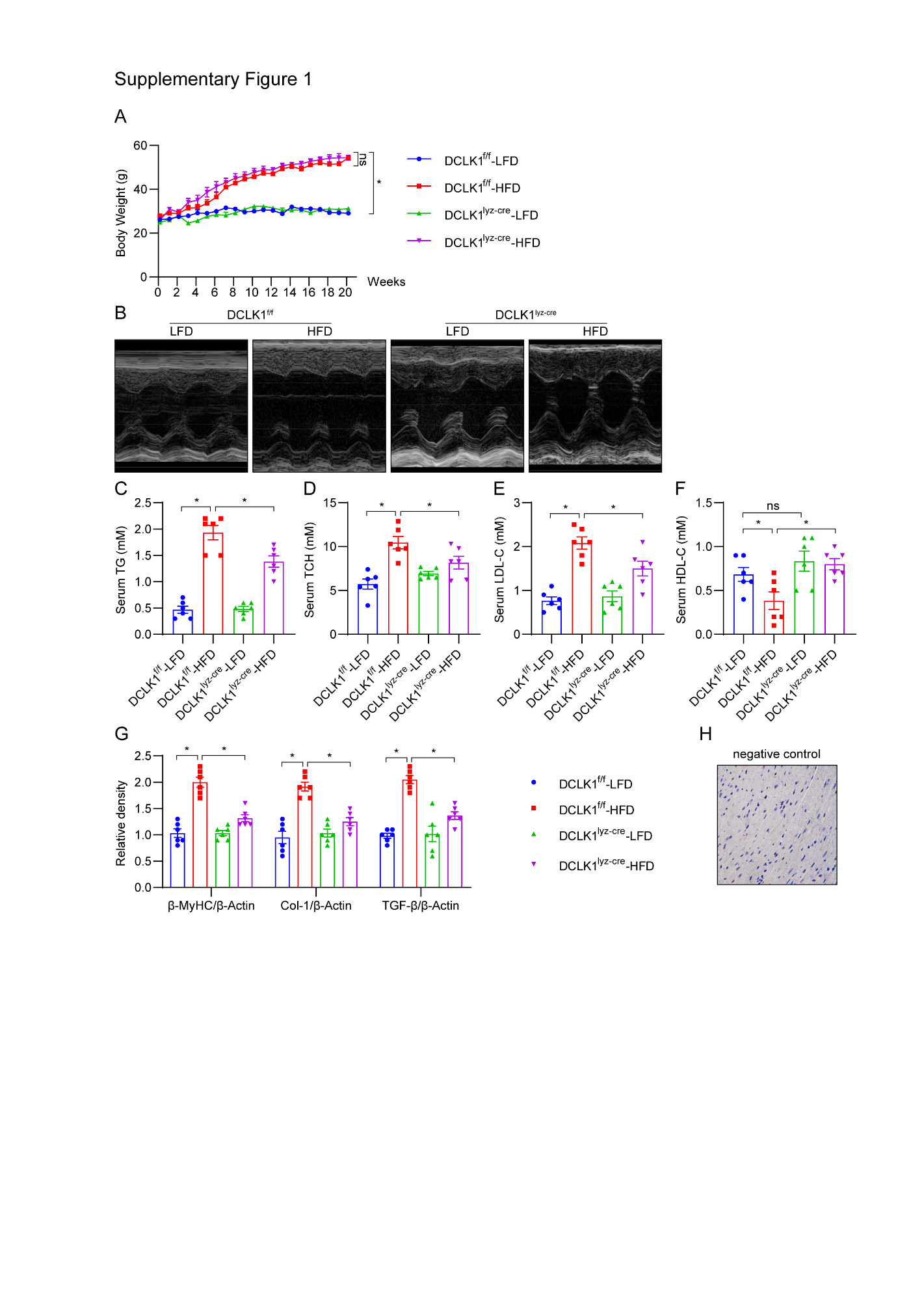


**Supplementary Figure S2: DCLK1 deficiency in macrophages does not alter body weight and reduces cardiac dysfunction of mice.**

Macrophage-specific DCLK1 knockout (DCLK1^lyz-cre^) mice were generated by crossing DCLK1 floxed mice (C57BL6/JGpt background) and mice with Lyz2-Cre knockin. Lyz2 served as a monocyte-macrophage specific promoter. DCLK1^lyz-cre^ and DCLK1^f/f^ (used as control) were fed an LFD or a HFD for 20 weeks. (A)Body weights were measured weekly [n = 6; one-way ANOVA followed by Tukey post-hoc test; Mean ± SEM; ns = not significant; *p<0.05]. (B) Representative echocardiographic images showing HFD-induced cardiac deficits [n = 6]. (C-F) Serum levels of heart function lipids [n = 6; one-way ANOVA followed by Tukey post-hoc test; Mean ± SEM; *p<0.05]. (G) Densitometric quantification of blots in Figure 2I. [n = 6; one-way ANOVA followed by Tukey post-hoc test; Mean ± SEM; *p<0.05]. (H) The negative control of Figure 2K.

**
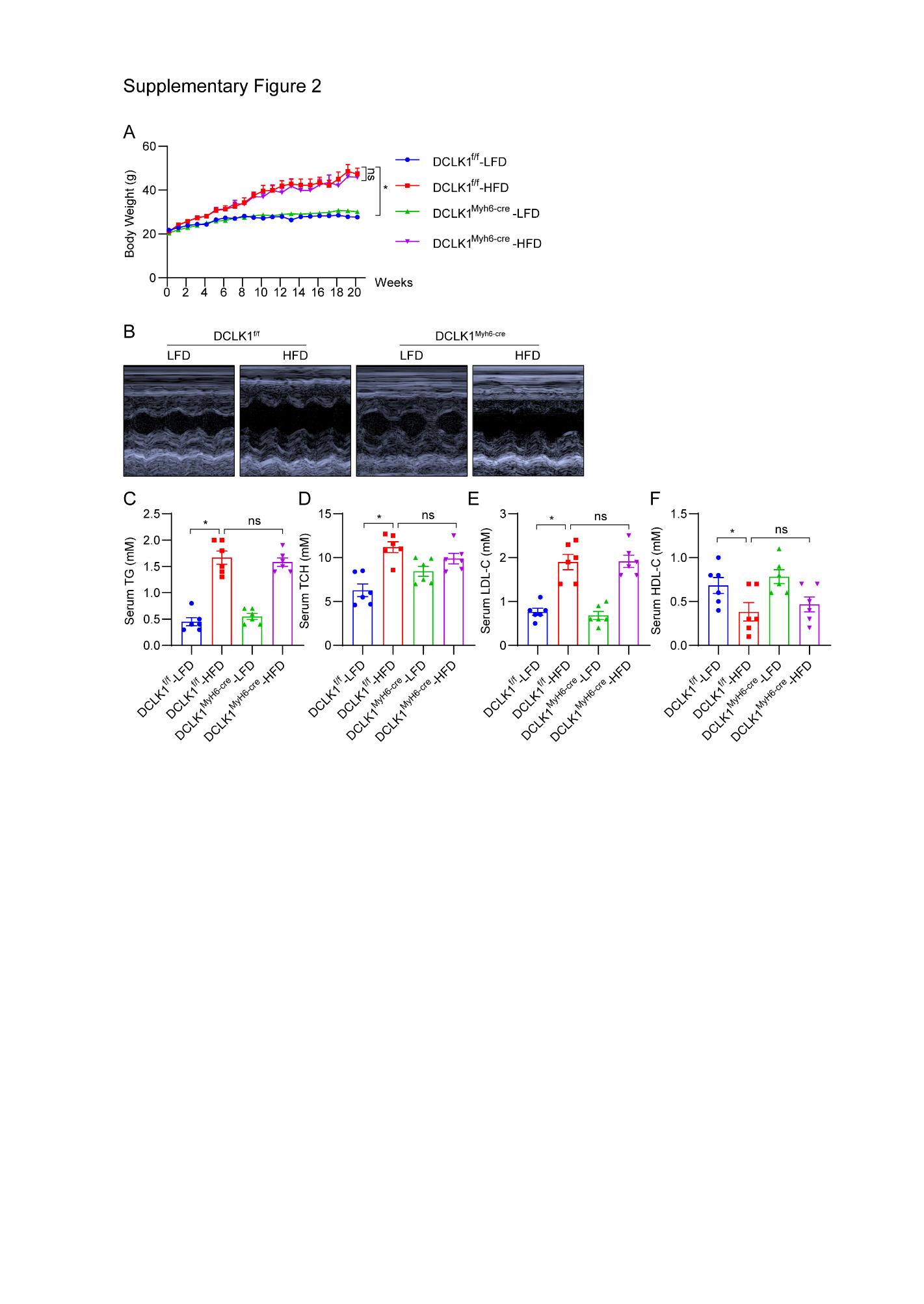
**

**Supplementary Figure S3:** **cardiomyocyte-specific DCLK1 knockout does not alter body weight and cardiac dysfunction of mice.**

The cardiomyocyte-specific DCLK1 knockout mice (DCLK1^Myh6-cre^) were generated using the Cre-loxP method. Mice floxed for DCLK1 (DCLK1^f/f^) were crossed with mice carrying Cre-transgene under the promoter of the Myh6 gene (Myh6-Cre) which led to the generation of DCLK1^Myh6-cre^ mice. DCLK1^MyH6-cre^ and DCLK1^f/f^ (used as control) were fed an LFD or a HFD for 20 weeks. (A)Body weights were measured weekly [n = 6; one-way ANOVA followed by Tukey post-hoc test; Mean ± SEM; ns = not significant; *p<0.05]. (B) Representative echocardiographic images showing HFD-induced cardiac deficits [n = 6]. (C-F) Serum levels of heart function lipids [n = 6; one-way ANOVA followed by Tukey post-hoc test; Mean ± SEM; ns = not significant; *p<0.05].

**
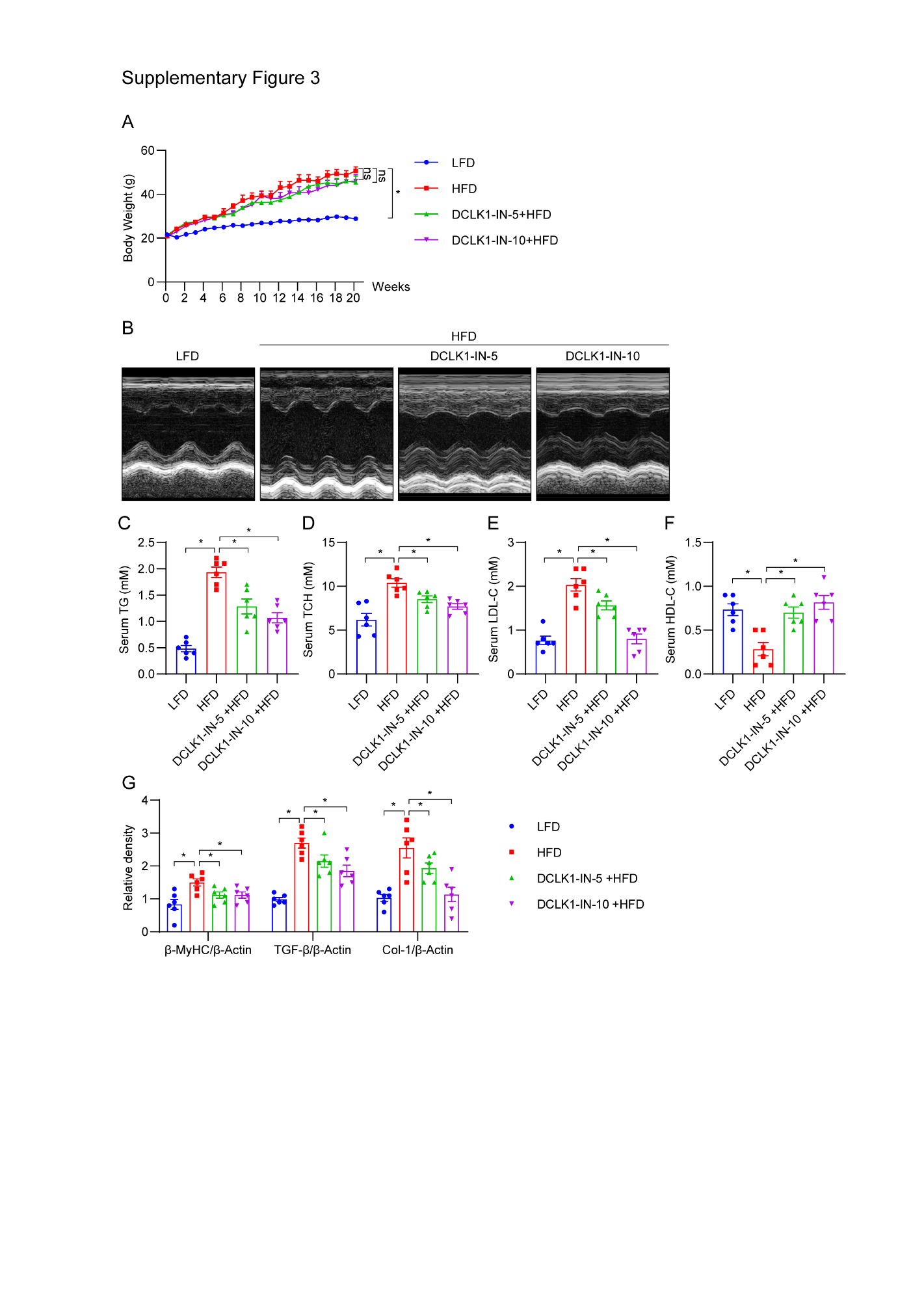
**

**Supplementary Figure S4: DCLK1 inhibitor does not alter body weight and reduces cardiac dysfunction of mice.**

(A)Body weights were measured weekly [n = 6; Mean ± SEM; ns = not significant; *p<0.05]. (B) Representative echocardiographic images showing HFD-induced cardiac deficits [n = 6]. (C-F) Serum levels of heart function lipids [n = 6; one-way ANOVA followed by Tukey post-hoc test; Mean ± SEM; *p<0.05]. (G) Densitometric quantification of blots in Figure 4I [n = 6; one-way ANOVA followed by Tukey post-hoc test; Mean ± SEM; *p<0.05].

**
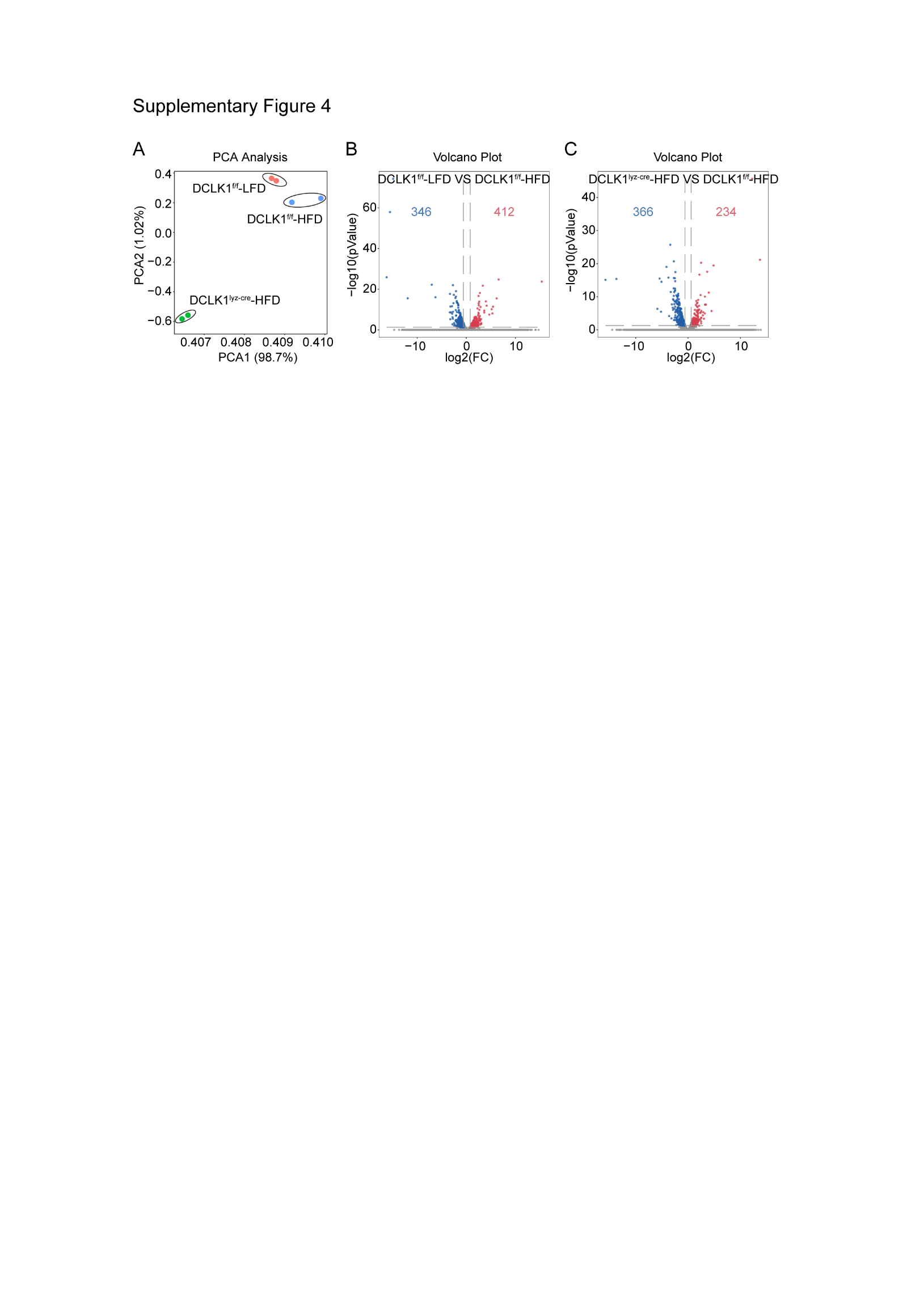
**

**Supplementary Figure S5: Detection of DCLK1-regulated pathways in HFD fed mice.**

(A) PCA analysis of RNA-sequencing data [n = 6; Mean ± SEM; *p<0.05]. (B, C) Volcano plot analysis of transcriptional changes in DCLK1^f/f^ -LFD compared to DCLK1^f/f^ -HFD (B), and DCLK1^lyz-cre^ -HFD compared to DCLK1^f/f^ -HFD [n = 6; Mean ± SEM; *p<0.05]. (C) The fold change threshold was set as 2 and P-value ≤ 0.01. Red dots indicate upregulated genes, blue dots indicate downregulated genes, and gray dots indicate non-differentially expressed genes [n = 6; Mean ± SEM; *p<0.05].

**
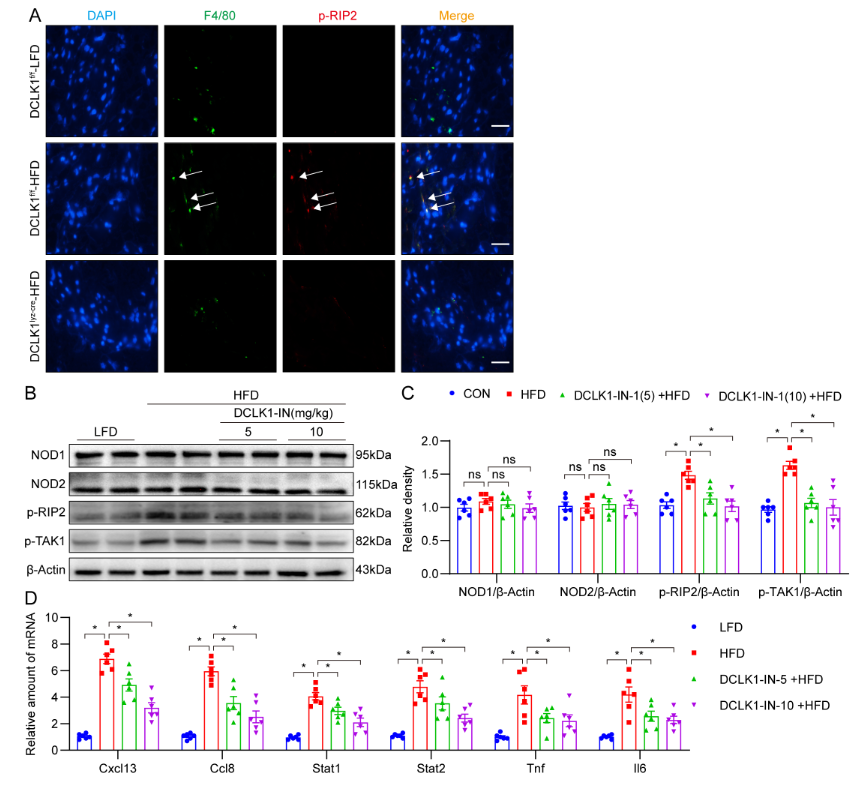
**

**Supplementary Figure S6: DCLK1 inhibitor** **alleviate HFD-induced myocardial injury by inhibiting RIP2/TAK1 signal pathway.**

(A) Representative immunofluorescence staining for F4/80 and p-RIP2 in heart tissues of mice from DCLK1^f/f^-LFD, DCLK1^f/f^-HFD, and DCLK1^lyz-cre^-HFD groups. Tissues were counterstained with DAPI (blue) [scale bar = 50 μm; n = 6]. (B) Representative Western blot analysis of NOD1, NOD2, p-RIP2 and p-TAK1 in the heart tissues. β-Actin was used as the loading control [n = 6; Mean ± SEM; *p<0.05]. (C) Densitometric quantification of blots in panel A [n = 6; one-way ANOVA followed by Tukey post-hoc test; Mean ± SEM; *p<0.05]. (D) The mRNA levels of *Cxcl13*, *Ccl8*, *Stat1, Stat2, Tnf and Il6* were detected by RT-qPCR in the heart tissues [n = 6; one-way ANOVA followed by Tukey post-hoc test; Mean ± SEM; *p<0.05].

**
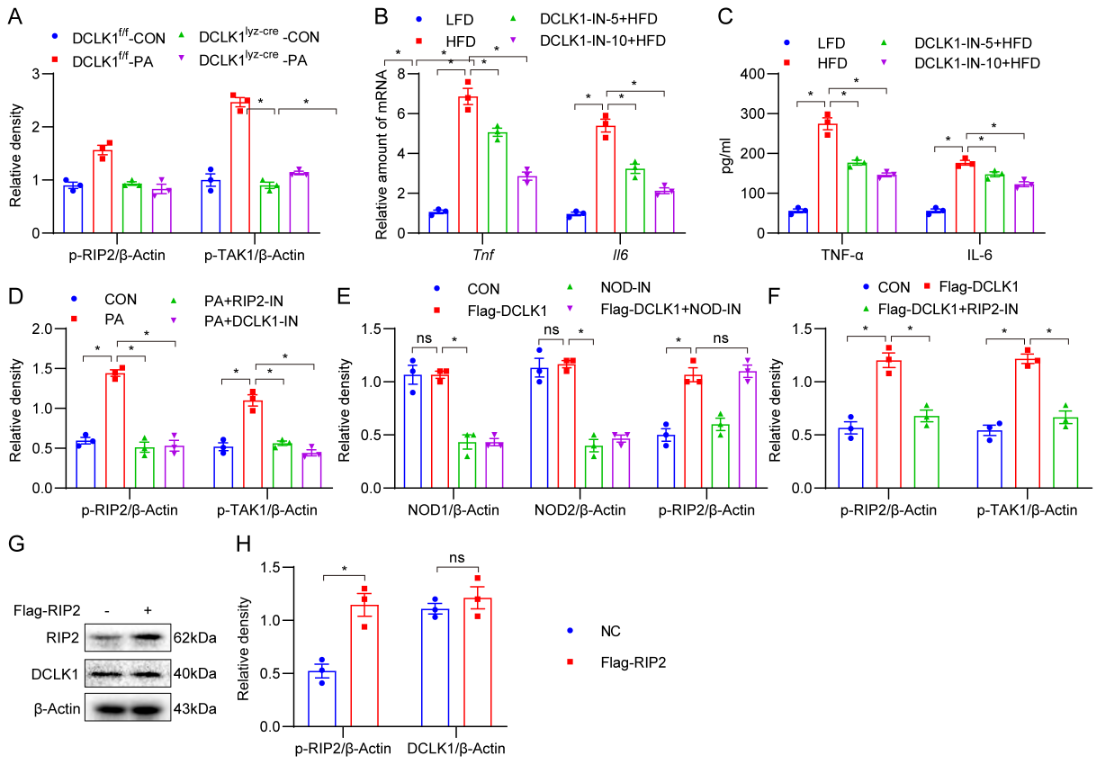
**

**Supplementary Figure S7: Deleting DCLK1 in macrophages inhibits PA-induced activation of the RIP2/TAK1 signal pathway and inflammatory responses.**

(A) Densitometric quantification of blots in Figure 6A [n = 3; one-way ANOVA followed by Tukey post-hoc test; Mean ± SEM; *p<0.05]. (B) The mRNA levels of *Tnf* and *Il6* were detected by RT-qPCR in the heart tissues [n = 3; one-way ANOVA followed by Tukey post-hoc test; Mean ± SEM; *p<0.05]. (C) MPMs were exposed to 200 μM PA or 10% BSA (con) for 12 h. Levels of TNF-α and IL-6 protein levels in culture media were determined [n = 3; one-way ANOVA followed by Tukey post-hoc test; Mean ± SEM; *p<0.05]. (D) Densitometric quantification of blots in Figure 6D [n = 3; one-way ANOVA followed by Tukey post-hoc test; Mean ± SEM; *p<0.05]. (E) Densitometric quantification of blots in Figure 6E [n = 3; one-way ANOVA followed by Tukey post-hoc test; Mean ± SEM; *p<0.05]. (F) Densitometric quantification of blots in Figure 6F [n = 3; one-way ANOVA followed by Tukey post-hoc test; Mean ± SEM; *p<0.05]. (G) Representative Western blot analysis of RIP2 and DCLK1 in the MPMs. β-actin was used as the loading control [n = 3; Mean ± SEM; *p<0.05]. (H) Densitometric quantification of blots in G [n = 3; Student’s t-test; Mean ± SEM; *p<0.05].


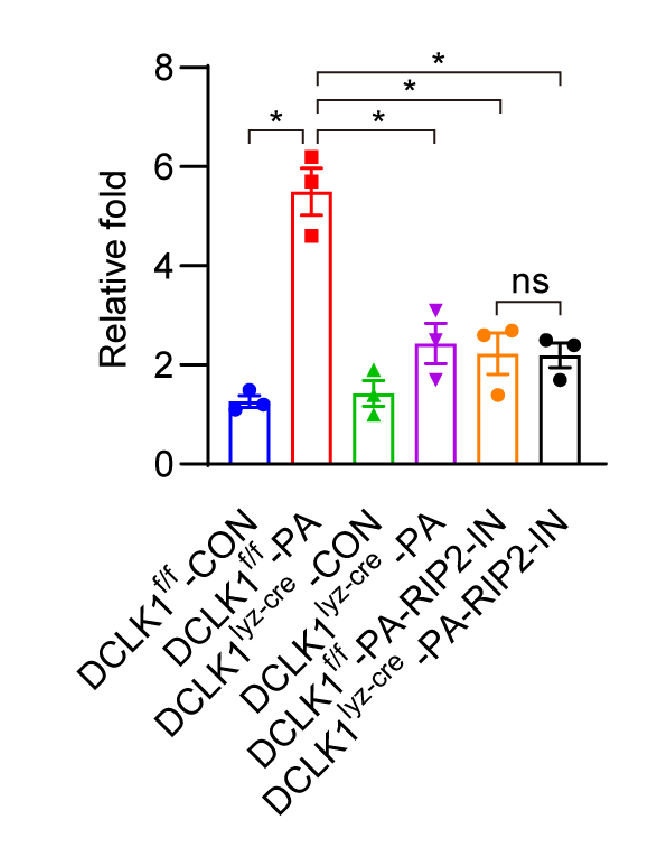


**Supplementary Figure S8:** Quantification of cell size in images in Figure 7B. A minimum of 100 cells were measured from different visual fields of 3 samples per group [n = 3; one-way ANOVA followed by Tukey post-hoc test; Mean ± SEM; *p<0.05].


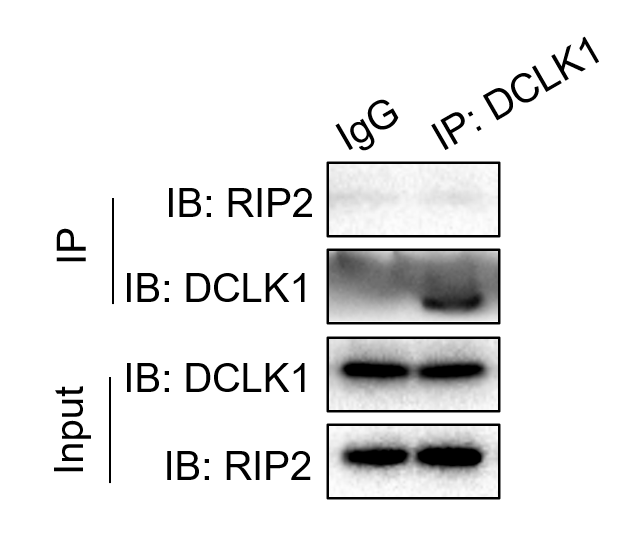


**New supplementary Figure S9:** DCLK1 has no direct interaction with RIP2 in macrophages. MPMs were challenged with 200 μM PA for 30 min. Lysates from cells were immunoprecipitated with anti-DCLK1 and anti-RIP2. RIP2 levels were detected by immunoblotting. IgG was used as control for IP assay.
